# Habitats, Plant Diversity, and Molecular Phylogeny of Endemic Relic Species *Incarvillea semiretschenskia* (Bignoniaceae)

**DOI:** 10.3390/plants13233299

**Published:** 2024-11-23

**Authors:** Liliya Dimeyeva, Valeriya Permitina, Alfiya Kurmantayeva, Azhar Imanalinova, Bektemir Osmonali, Farida Kozybayeva, Gulzhan Beiseyeva, Kapar Ussen, Rashid Iskakov, Batlai Oyuntsetseg, Nikolai Friesen

**Affiliations:** 1 Institute of Botany and Phytointroduction, 36D Timiryazev Str., Almaty 050040, Kazakhstan; v.permitina@mail.ru (V.P.); kurmanalfia@mail.ru (A.K.); azhar.imanalinova@gmail.com (A.I.); be96ka_kz@mail.ru (B.O.); ussen.kapar@mail.ru (K.U.); poligon2008@mail.ru (R.I.); 2 Uspanov Kazakh Research Institute of Soil Science and Agrochemistry, 75B al-Farabi av., Almaty 050060, Kazakhstan; farida_kozybaeva@mail.ru (F.K.); beiseeva2009@mail.ru (G.B.); 3 Department of Biology, School of Arts and Sciences, National University of Mongolia, Ulaanbaatar 14201, Mongolia; oyuna62@yahoo.com; 4 Botanical Garden, University of Osnabrück, 29 Albrechtstrasse, 49076 Osnabrück, Germany; nfriesen@uni-osnabrueck.de

**Keywords:** *Incarvillea*, DNA molecular analysis, Shu-Ile low mountains, ecological conditions, floristic composition, nature conservation

## Abstract

*Incarvillea semiretschenskia* (B. Fedtsch.) Grierson is listed in the Red Data Book of Kazakhstan as a rare relic, narrowly endemic species of the Shu-Ile low mountains (Kazakhstan). The aim of this research was to advance our knowledge of the ecological conditions of its habitats, the floristic composition of plant communities, and molecular phylogeny, as well as to identify threats to the species’ existence. The ecological conditions of *I. semiretschenskia* habitats are rocky slopes and intermountain valleys of the low mountains in the altitude range from 812 to 1075 m asl with light chestnut mountain soils of little development, having a light granulometric composition and containing insignificant amounts of organic matter. We revealed 164 species of vascular plants in the communities of *I. semiretschenskia*, including five endemics and five species from the Red Data Book of Kazakhstan. Anthropogenic factors associated with grazing, fires, and limestone mining were identified as leading to a decrease in the number and density of populations. New insights into the relationship and time of diversification in the genus *Incarvillea* are gained through the use of several accessions of *I. semiretschenskia* and *I. potaninii* Batalin in the phylogenetic study. Preliminary fingerprint analysis shows relatively high genetic variability within populations of *I. semiretschenskia*. This unique relic species has survived since the Miocene epoch and exists to this day only in the Shu-Ile low mountains. To preserve this rare species, measures are proposed to create plant micro-reserves to provide ex situ collections and ensure future in situ restoration efforts.

## 1. Introduction

Species of the genus *Incarvillea* from the tropical family Bignoniaceae are mainly distributed in Southeast Asia, but several species have isolated distributions in the Gobi Altai (*I. potaninii* Batalin), in Kyrgyzstan (*I. olgae* Regel), and in the Shu-Ile mountains in Kazakhstan (*I. semiretschenskia* (B.Fedtsch.) Grierson). The history of the study of the genus *Incarvillea* is described in sufficient detail in the monograph by Winterholler [1]. According to one of the latest classifications of the genus *Incarvillea*, which includes eighteen species: *I. altissima* Forrest, *I. arguta* Royle, *I. beresowskii* Batalin, *I. compacta* Maxim., *I. delavayi* Bureau et Franch., *I. dissectifoliola* Q.S.Zhao, *I. emodi* Wall, *I. forrestii* H.R. Fletcher, *I. himalayensis* Grey-Wilson, *I. lutea* Bureau et Franch., *I. mairei* (H. Lev.) Grierson, *I. olgae, I. potaninii, I. semiretschenskia*, *I. sinensis* Lam., *I. uniflora* H.P. Deng et Chang Y. Xia, *I. younghusbandii* Sprague, and *I. zhongdianensis* Grey-Wilson. Grierson [2] divided the genera into five subgenera: *Amphicome*, *Olgaea*, *Incarvillea*, *Niedzwedzkia*, and *Pteroscleris*. The monotypic subgenus *Niedzwedzkia* with *I. semiretschenskia*.

*Incarvillea semiretschenskia* is distributed only in the Shu-Ile low mountains in Kazakhstan. *I. semiretschenskia* is listed in the Red Data Book of Kazakhstan as a rare relic, narrowly endemic species [3].

This species was first collected in 1909 during an expedition led by V. E. Niedzvedzky. The herbarium of collected samples was sent to the Imperial Botanical Garden in St. Petersburg. Only in 1915 was the plant described by B. A. Fedchenko [4] as *Niedzwedzkia semiretschenskia* B. Fedtsch. In revising the genus *Incarvillea*, A. Grierson [2] dissolved the genus *Niedzwedzkia* and placed it in the genus *Incarvillea* in its own subgenus *Niedzwedzkia*. Since then, it has been undisputed that *Niedzwedzkia semiretschenskia* belongs to the genus *Incarvillea*.

**Figure 1:**
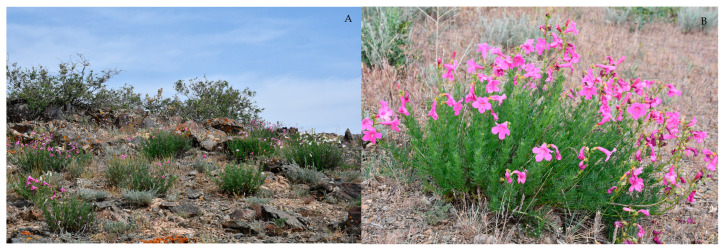
Community of *Incarvillea semiretschenskia* (**A**). The species in its habitat (**B**).

*I. semiretschenskia* (Figure 1A,B) is a xerophytic dwarf semishrub up to 70 cm high, 30–35 cm on average, with numerous woody, leafy stems at the base and leaves pinnately dissected into narrow, linear lobes. The flowers are regular on the top of the shoots. The corolla is bright pink, tubular-funnel-shaped, and 35–42 mm long with a 20–30 mm long tube and a 5–8 mm long bend. It has five rounded lobes, four stamens and fruit, with a capsule 30–40 mm long and 15 mm wide, with four to six winged, serrated ribs. Its ovoid seeds are 6–7 mm long. It is distributed in the slopes of the Shu-Ile low mountains, the Anarkhai upland, Aiderke, the Kopalysai tracts, and the Sarybulak and Aschisu interfluves. The plant blooms in May, with fruiting in June–July. It is propagated by seeds, in culture by dividing the bush and green cuttings [1,5,6,7,8,9,10,11].

According to the iNaturalist website [12], the species in Kazakhstan is marked at five locations and 130 points. In the Global Biodiversity Information Facility Secretariat checklist dataset [13], there are 50 results, 15 herbarium specimens (Moscow, Paris, Edinburgh, etc.) and 3 samples in live collections in Uzbekistan and SysTax—Botanical Gardens [13]. Notably, eight specimens of this species are held at the herbarium of the Institute of Botany and Phytointroduction (AA).

This plant has consistently aroused scientists’ interest through its study and has been described in detail in a book by B. Winterholler [1], an expert on the species who studied it in nature and culture from 1974 to 2003. We note that the locations of this species in nature have been repeatedly lost and rediscovered. There is still no complete picture of its distribution within the Shu-Ile low-mountain massif. The reduction in the distributional range and the impact of anthropogenic factors are alarming, leading to a decrease in the species diversity of communities and increasing the abundance of weeds [5].

**Figure 2:**
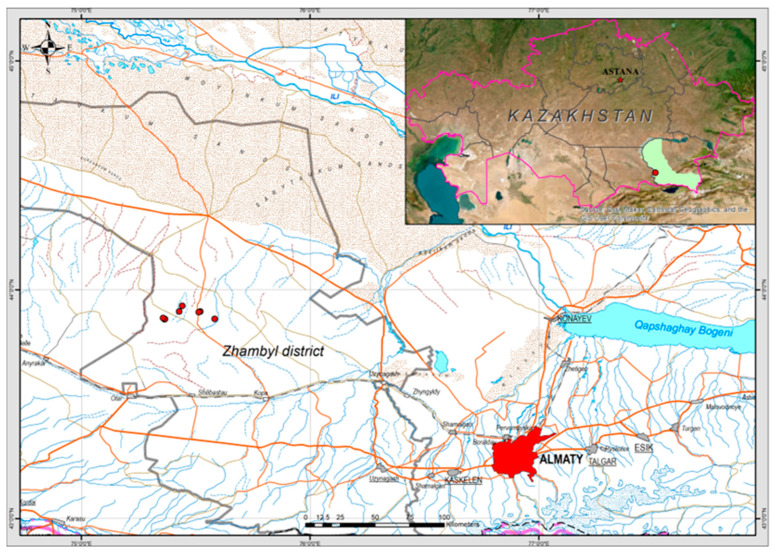
Distribution of *I. semiretschenskia*. The red dots show the distribution of sample plots in the study area. Location of the Almaty region is marked by green color in the Map of Kazakhstan.

The studies were carried out in the Shu-Ile low mountains in Kazakhstan. The research area is located within the Zhambyl district of the Almaty region (Figure 2).

The Shu-Ile low mountains are represented by arid denudation lowlands (1294 m above sea level (asl), with a dissection depth of about 100–400 m) with flat peaks and gentle slopes composed of Proterozoic and Lower Paleozoic gneisses, which are covered by crystalline shales with interlayers of marls belonging to the Silurian, broken through by granite intrusions. Conglomerate sandstones and limestones lie above. Tertiary gypsiferous clays come to the surface in the valleys between the hills. Current friable rocks are poorly sorted and of low thickness and are represented by rubble, gruss eluvium, proluvial deposits in valleys, and coarse deluvium at the foot of slopes. These mountains are dissected by a system of faults into raised blocks and depressions of different heights. The surface of the mountains is levelled by the processes of an ancient peneplain, with a pronounced alternation of gentle, undulating hillocks interspersed with shallow valleys. The mountain peaks have rocky ridges. As a result of the intensive destruction of rocks, the mountain spurs, represented by short and narrow ridges with signs of erosional dissection, are buried among the destruction products. The northern foothills of the Shu-Ile Mountains are characterized by undulating ridge relief with some lowlands with absolute altitudes of 500–800 m asl.

The Shu-Ile low mountains are distinguished by the poorly expressed altitudinal zonality of their landscapes due to differences in slope exposure and steepness, lithological heterogeneity, and fragmentary altitudinal belts of soil cover. This includes desert steppes in the lowlands with the development of mountain light chestnut soils on plateau-like peaks; mountain steppe deserts with light gray, often rubbly soils on undulating foothill plains; and brown desert soils on gentle northern slopes of mountains formed on proluvial, rubbly flint deposits. Bottoms of flat hollows with shallow groundwater (2–3 m) are occupied by meadow soils. Saline soils develop in relief depressions and along dry channels on the outcrops of Tertiary clays. Separately located low mountains are characterized by the formation of gray, crushed, stone mountain soils [14,15].

The climate is sharply continental. According to the Otar meteorological station, the average annual precipitation is 316 mm, the average annual air temperature is +7.9 °C, the average monthly temperature in January is −15.4 °C (the absolute minimum is −38 °C), the average monthly temperature in July is +32.7 °C (the absolute maximum is +43 °C), and the sum of air temperatures above 10 °C is 3116 °C [16].

Our field research was conducted in 2023 to describe rare plant communities of the Almaty region [17]. We knew of only one habitat of the species in the Tyrnakty Important Plant Area [18], which we found. We searched for new locations according to the descriptions on the herbarium labels. We found another site thanks to a herbarium specimen collected by Kubanskaya Z. V. in 1936 on a limestone ridge between the Sarybulak and Aschisu rivers located in the Shilozek tract. The limestone quarry activities in the site have destroyed one slope of the limestone ridge with *I. semiretschenskia*. There is a danger of completely losing a rare population with the further continuation of mining operations.

There are only a few phylogenetic publications with DNA analyses of the genus *Incarvillea* by Chinese authors [19,20,21], with nrITS and trnL-trnF sequences from 14 *Incarvillea* species. In these works, on the nrITS tree, the accession of *I. semiretschenskia* is sister to all species of the genus *Incarvillea* and is a sister to *I. olgae* Regel. In the plastid trnL-trnF tree, however, *I. semiretschenskia* stands in the middle of the tree and is a sister to *I. sinensis* Lam. [21]. Rana et al. [21] also presented the dating of the genus *Incarvillea*, based on trnL-trnF ICS. The molecular dating analyses suggest that *Incarvillea* might have evolved before the Miocene during the mid-Oligocene, ca. 19.09 ± 3.89 Ma.

Unfortunately, an important *Incarvillea* species from Mongolia, *I. potaninii*, is missing in these analyses. In the publications of Chen et al. [19,20], the distribution of *I. semiretschenskia* is also incorrectly given for Kyrgyzstan. Our work closes this gap with several accessions of *I. semiretschenskia* and *I. potaninii*.

The aim of this research was to advance our knowledge of the endemic relic species *I. semiretschenskia*. The tasks undertaken to achieve this goal are as follows: (1) to reveal landscape and soil conditions of its habitats; (2) to analyze the data on the floristic composition of plant communities; (3) to close the gap in the molecular phylogeny of *I. semiretschenskia* and *I. potaninii*; and (4) to identify threats to the species’ existence and find ways for its conservation.

## 2. Results

### 2.1. Ecological Conditions and Vegetation Cover

#### 2.1.1. Tyrnakty Site (B)

##### Habitats Dominated and Co-Dominated by *I. semiretschenskia*

The habitats dominated and co-dominated by *I. semiretschenskia* are intermountain valleys with ridges and outcrops of effusive rocks in the form of domes of oval or flat shape, occupying up to 80%, as well as hillsides of south-eastern and eastern exposure, with a 25° slope (1063 m asl.). The thickness of the stony cape is up to 5–10 cm. The vegetation comprises ephemerides–sagebrush–Inkarvillea with shrubs (total projective coverage, TPC: 35–40%), with a shrub layer (projective coverage, PC: 6–7%): *Atraphaxis pyrifolia* Bunge, *Ephedra intermedia* Schrenk & C.A. Mey., *Prunus griffithii* var. *tianshanica* (Pojark.) Ingram, *Spiraea hypericifolia* (Pojark.) Ingram, dwarf semishrub layer (PC: 7–10%): *Artemisia juncea* Kar. & Kir., *A. sublessingiana* (B.Keller) Krasch. ex Poljakov, *I. semiretschenskia*, *Bassia prostrata* (L.) Beck, and a herbal layer (PC: 25–35%): *Allium petraeum* Kar. & Kir., *Alyssum desertorum* Stapf, *Astragalus schrenkianus* Fisch. & C.A. Mey., *A. chaetodon* Bunge, *Biebersteinia multifida* DC., *Bromus tectorum* L., *Eremurus cristatus* Vved., *Eremopyrum orientale* (L.) Jaub. & Spach, *Festuca valesiaca* Schleich. ex Gaudin, *Goniolimon cuspidatum* Gamajun., *Haplophyllum acutifolium* (DC.) G. Don, *Iris kuschakewiczii* B. Fedtsch., *Ixiolirion tataricum* (Pall.) Schult. & Schult. f., *Minuartia meyeri* (Boiss.) Bornm., *Poa bulbosa* L., *Potentilla soongorica* Bunge, *Rosularia glabra* (Regel & C. Winkl.) A. Berger, *Schrenkia involucrata* Regel & Schmalh., *Secale sylvestre* Host, *Seseli sessiliflorum* Schrenk, *Sibbaldianthe bifurca* (L.) Kurtto & T. Erikss., *Stipa sareptana* A.K. Becker, *Tragopogon marginifolius* Pavlov, *Tulipa biflora* Pall., and *Ziziphora tenuior* L.

The species is also found in intermountain hilly and ridging plains, slopes of hills and ridges with rock outcrops and a stony cape on the surface, and in lower areas complicated by effusive rock outcrops in the form of domes of convex or flat shape (1074–1075 m asl).

The vegetation includes ephemeroid–bulbous bluegrass–fescue–Inkarvillea (*I. semiretschenskia*, *Festuca valesiaca*, *Poa bulbosa*) with shrubs on the west and south-west slopes and grass–shrub–Inkarvillea on the gentle northern slopes (TPC: 40%), with a shrub and dwarf shrub layer (PC: 10–15%): *Helianthemum songaricum* Schrenk ex Fisch. & C.A.Mey., *Spiraea hypericifolia*, *Prunus griffithii* var. *tianshanica*, dwarf semishrub layer (PC: 5–10%): *I. semiretschenskia* and a herbal layer (PC: 15%): *Alyssum desertorum*, *Astragalus schrenkianus*, *Eremurus cristatus*, *Festuca valesiaca*, *Gentiana olivieri* Griseb., *Haplophyllum acutifolium*, *Iris kuschakewiczii*, *Poa bulbosa*, *Potentilla soongorica*, *Pseudosedum affine* (Schrenk) A. Berger, *Ranunculus testiculatus* Crantz, and *Tragopogon ruber* S.G. Gmel.

Soils: The light chestnut mountain soils are underdeveloped, occupy dissected surfaces, and are confined to peaks and slopes of mountains. They are often interrupted by rock outcrops, develop on the eluvium of rocks, and are strongly rubbly (skeletal). They are distinguished by their shortened profile (15–25 cm) with an insignificant fine-earth layer, which is formed by the boiling of hydrochloric acid on the surface. According to the conditions of the relief and constituent rocks, they represent xeromorphic soils with weak manifestations of soil formation processes. In the upper part of the profile, there is a schistosity horizon (0–1.5 cm) of a dark, brownish-gray color, loose composition, and dusty–powdery structure, passing to the pale-brown horizon of a slightly compacted composition, with a dusty, loose, and lumpy structure. Below, there is a layer of crushed stone with brown-colored, fine-grained sand, occupying cavities and cracks between stony fragments, which is underlaid by a layer of bedrock fragments. Soils in the 0–15 cm layer are characterized by a very low humus content (no more than 1.52%) and low total nitrogen content (0.112%). The carbon-to-nitrogen ratio (C:N = 7.9) is narrow, and the provision of soils with nitrogen of hydrolysable compounds is low (50.4 mg/kg). The provision of soils with mobile forms of phosphorus is high (37 mg/kg), and that with mobile potassium forms is average (300 mg/kg). The soils are carbonaceous and contain 2.2% carbonates. The reaction of the soil solution is alkaline, with pH = 8.8. The absorption capacity (on the sum of absorbed bases) is low, and compounds comprise 14.14 mg-eq per 100 g of soil; this is caused by a low content of silty fractions and humus, the amount of which depends on the value of ions capable of exchange. The absorption complex of the horizon is saturated with calcium cations (69% of the total absorbed bases), with the significant participation of magnesium cations (25% of the total). Exchangeable sodium comprises up to 3.4% of the total, and the soils are not saline. The soils are not salted with easily soluble salts, and the sum of salts does not exceed 0.057%. According to their granulometric composition, the soils are light and loamy, with a predominance of fine sand (53.66%) and silty fractions (16.60%). The predominance of sandy fractions (72.00%) in the granulometric composition was revealed and used to determine surface passivity with respect to the absorption capacity of soils, increased filtration capacity, and good aeration [22].

##### Habitats with Sparse Distribution of *I. semiretschenskia*

The habitats with a sparse distribution of *I. semiretschenskia* are intermountain valleys, with an elevated rise in the form of a rampart orientated parallel to the slope bottom with rock outcrops, a slope of south-western exposure and an incline of 15° (1078 m asl). The surface is covered with a layer of bedrock debris.

The vegetation is sagebrush–ephemeroid–Helianthemum (*Helianthemum songaricum*, *Artemisia sublessingiana*, *A. juncea*) (TPC: 30%), with a dwarf shrub layer (PC: 15%): *H. songaricum*, *Ephedra intermedia*, semishrub and dwarf semishrub layers (PC: 5%): *Artemisia sublessingiana*, *I. semiretschenskia* and a herbal layer (PC: 10%): *Alyssum desertorum*, *Astragalus schrenkianus*, *A. chaetodon*, *Biebersteinia multifida*, *Bromus tectorum*, *Eremurus cristatus*, *Euphorbia rapulum* Kar. & Kir., *Centaurea virgata* subsp. *squarrosa* (Boiss.) Gugler, *Goniolimon speciosum*, *Haplophyllum acutifolium*, *Iris halophila* var. *sogdiana* (Bunge) Skeels, *I. kuschakewiczii*, *Ixiolirion tataricum*, *Lagochilus platycalyx* Schrenk ex Fisch. & C.A. Mey., *Poa bulbosa*, *Oedibasis apiculata* (Kar. & Kir.) Koso-Pol., *Ranunculus testiculatus*, *Scutellaria titovii* Juz., *Stipa orientalis* Trin., *S. richteriana* Kar. & Kir., and *Ziziphora tenuior.*

Soils: The light chestnut mountain soils are incompletely developed and formed on the eluvium of dense rocks. The surface is covered with a layer of rock fragments. The soil profile is low in thickness, 50–55 cm, strongly stony, and is formed by the boiling of hydrochloric acid on the surface. In the upper part, there is a layered horizon of brownish-gray color and a loose dusty–powdery structure, overlapped by a thin layer of dark gray color and passing into a pale-brown, compacted horizon of loose, lumpy–powdery structure. Below, there is a light-brown carbonaceous horizon of loose, lumpy structure and dense composition with carbonates in the form of vague, whitish spots in the lower part and crushed stone inclusions with carbonaceous crusts on the lower surface of the separate parts. This area is underlain by a layer of bedrock fragments with a small amount of fine earth in the cavities and cracks. The humus content in the upper (0–7 cm) horizon is very low, no more than 1.45%, and decreases with depth to 0.7% with a low content of total nitrogen (0.07–0.112%). The carbon-to-nitrogen ratio is narrow (C/N = 6.0–7.5). The provision of soils with nitrogen of hydrolysable compounds is medium and low (42–56 mg/kg), and the provision of soils with mobile forms of phosphorus is low and very low (3–18 mg/kg), while the provision of mobile forms of potassium is medium and low (70–260 mg/kg). The soils are carbonaceous from the surface, and the carbonate content is 8.6%. With an increase in depth, the quantity of carbonates increases to 12.5–13.1%. The reaction of the soil solution is alkaline (pH = 8.5–8.9). The absorption capacity (on the sum of absorbed bases) is low and does not exceed 10.67–13.83 mg-eq per 100 g of soil. Calcium cations prevail in the composition of absorbed bases (49–74% of the total absorbed bases), with significant participation of magnesium cations (19–45% of the total). The sodium cation share is up to 3.7% of the total, and the soils are not saline. The soils are not salted with easily soluble salts, and the sum of salts does not exceed 0.228%. According to their granulometric composition, the soils are light and loamy, with a predominance of fine sand (33.08%) and coarse dust (25.41%) fractions. In the distribution of silt–dust fractions along the profile, equal contents at all depths are observed (20.57–23.48%). The redistribution of the silt fraction (from 5 to 13%), with a formation of illuvial horizon and a sharp decrease in silt values up to 4.5% in the lower horizon, is expressed in the soil profile. An increase in the fine sand fraction up to 60% and a significant content of the medium sand fraction with an insignificant variation in physical clay particles are noted with depth, which determines the soil’s low absorption capacity, characterizes the low water-holding capacity of soil horizons, and increases filtration capacity [22].

#### 2.1.2. Shilozek Site (A)

The Shilozek site consists of foothill, hilly ridges, strongly dissected plains (true altitude 812 m asl), and hill slopes with signs of erosion. The surface is complicated by outcrops and fragments of calcareous rocks. Soil-forming rocks are proluvial deposits overlain by loess-like loams with a limestone admixture.

The vegetation is ephemeroid–bunch grass–Inkarvillea (TPC: 25–30%) along the tops and upper part of the hills of the northern and north-eastern exposures, with a slope of 25–30° and a shrub layer (PC: 3–5%): *Atraphaxis spinosa*, *Spiraea hypericifolia*, *Bassia prostrata*, semishrub and dwarf semishrub layers (PC: 10–20%): *Artemisia sublessingiana*, *Krascheninnikovia ceratoides* (L.) Gueldenst., *I. semiretschenskia* and a herbal layer (PC: 15–20%): *Allium petraeum* Kar. & Kir., *Artemisia juncea*, *Astragalus chaetodon*, *Bromus tectorum*, *Eremopyrum orientale*, *Eremurus cristatus*, *Euphorbia rapulum*, *Festuca valesiaca*, *Haplophyllum acutifolium*, *Iris songarica* Schrenk ex Fisch. & C.A. Mey., *I. kuschakewiczii*, *Jurinea adenocarpa* Schrenk ex Fisch. & C.A. Mey., *Lagochilus platycalyx*, *Poa bulbosa*, *Potentilla soongorica*, *Rindera tetraspis* Pall., *Seseli sessiliflorum*, *Stipa caucasica* Schmalh., *S. conferta* Poir., *S. hohenackeriana*, *S. orientalis*, and *Tragopogon ruber.*

Soils: The soil cover is represented by mountainous, light chestnut, underdeveloped, crushed stone soils developing on the eluvium of calcareous rocks. The soils are characterized by a weakly formed profile containing a significant amount of crushed stone. The thickness of the soil profile does not exceed 25–30 cm and is formed by the boiling of hydrochloric acid on the surface. In the upper part of the profile, there is a light gray horizon of a loose formation of dusty–powdery structure, passing into a whitish-brown horizon of a compacted formation of a powdery–softly lumpy structure. Below, there is a horizon of accumulation of carbonate excretions of light-brown color of compacted composition underlain by a layer of limestone fragments and limestone rocks. The soils contain up to 1.5% humus in the surface horizon. They are not saline, are light and loamy in granulometric composition, and are characterized by an increased carbonate content reaching 13–15%.

### 2.2. Floristic Composition of Plant Communities

There are 85 species of vascular plants belonging to 28 families and 59 genera in the surveyed areas (Table 1). The leading families are Poaceae (17 species), Asteraceae (9), Lamiaceae (7), Apiaceae (6), and Rosaceae (5), which accounted for 36% of the plant communities’ flora. The leading genera are *Stipa*, nine species; *Iris*, four species; and *Astragalus*, *Artemisia,* and *Atraphaxis*, three species each. There are three endemics of Kazakhstan: *I. semiretschenskia*, *Phlomoides septentrionalis* (Popov) Adylov, Kamelin & Makhm., and *Schrenkia involucrata*. Three species are listed in the Red Data Book of Kazakhstan [3]: *I. semiretschenskia*, *Iris kuschakewiczii*, and *Tulipa biflora*.

**Figure 3:**
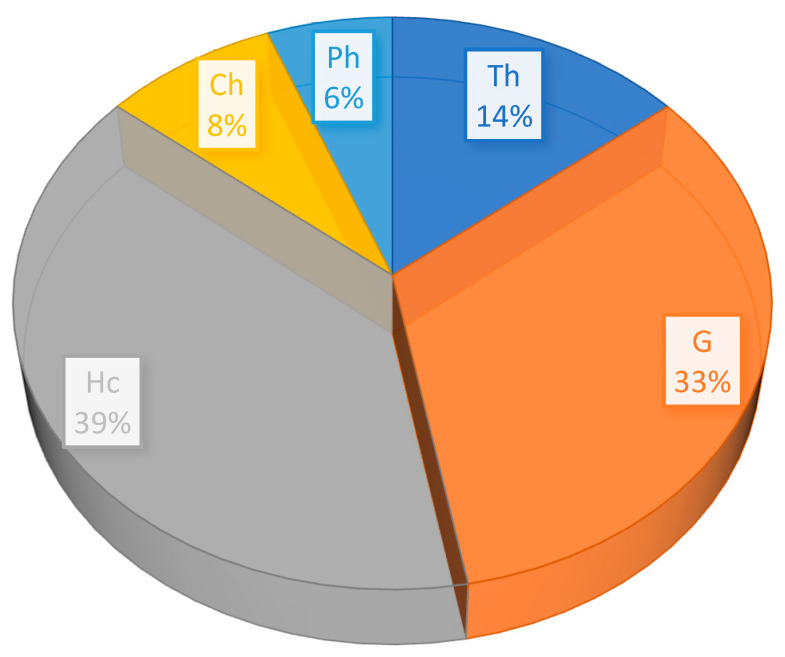
The spectrum of life forms: hemicryptophytes (Hc), geophytes (G), therophytes (Th), chamaephytes (Ch), phanerophytes (Ph).

The spectrum of life forms is dominated by herbaceous forms (86%): perennials (61 species) and annuals (12 species). According to the Raunkiaer classification [23], the most common group is hemicryptophytes (Hc) (33; 38.8%): *Bothriochloa ischaemum*, *Centaurea virgata* subsp. *squarrosa*, *Festuca valesiaca*, and *Haplophyllum acutifolium*, with species of the genus *Stipa*, among others (Figure 3). The group of geophytes (G) (28 species; 33%) consists of ephemeroids with bulbs that are species of the genus *Allium*, as well as *Ixiolirion tataricum*, *Tulipa biflora*. Those with rhizomes/roots or tubers are species of the genus *Iris*, along with *Biebersteinia multifida*, *Gelasia circumflexa*, *Ranunculus platyspermus*, *Rindera tetraspis*, and so on. The group of therophytes (Th) (12 species; 14.1%) is mainly represented by ephemerals, such as *Alyssum desertorum*, *Bromus tectorum*, *Eragrostis minor*, *Meniocus linifolius*, *Minuartia meyeri*, *Ranunculus testiculatus*, and so on. The chamaephytes (Ch) (7; 8.2%) group consists of semishrubs, dwarf shrubs, and dwarf semishrubs with an annual dying-off of the generative shoots. The Ch group is the most characteristic group for the arid zone of Central Asia and is associated with the influence of drought and the ability to survive in the dry summer season [24]. It includes species of the genus *Artemisia* as well as *Bassia prostrata*, *Helianthemum songaricum*, *I. semiretschenskia*, and *Krascheninnikovia ceratoides.* Phanerophytes (Ph) (5; 5.9%) are represented by shrubs (nanophanerophytes), such as *Atraphaxis compacta*, *A. pyrifolia*, *A. spinosa*, *Prunus griffithii* var. *tianshanica*, and *Spiraea hypericifolia*.

A comparison of the floristic lists of the two growing sites showed that the Sörensen coefficient was 0.30. This indicates differences related to environmental conditions and degrees of anthropogenic impact.

The projective coverage of *I. semiretschenskia* in communities does not exceed 10–15%. The size of populations in the Turnakty site varies from 832 to 16,452 sq. m, and the density on the plot of 100 sq. m varies from 16 to 58 plants. In the Shilozek site, the perimeter of the limestone ridge is 1615 m. The ridge is oriented from the north-east to the south-west. The *I. semiretschenskia* population has been preserved on the north-east slope, where the projective coverage is no more than 5–7%, and the population density per 100 sq. m is 54–109 plants.

To compile a general floristic list of *I. semiretschenskia* communities, we used all available literary sources [1,6,7,8,18,25,26,27,28,29,30]. We added the species that we identified ourselves (Table 1) to the resulting list. The result was a list of species of *I. semiretschenskia* communities (Table S1).

In total, 164 species of vascular plants belonging to 33 families and 94 genera have been identified in the communities of *I. semiretschenskia*. The leading families are Amaranthaceae (21 species), Asteraceae (18), and Brassicaceae (11), as well as Rosaceae, Fabaceae, and Poaceae (10 species in each). The leading genera are *Stipa* (10), *Allium* (8), *Astragalus* (7), and *Artemisia* (5). The generalized data allowed us to expand the ideas obtained from point studies in two habitats and add 79 more species to our list (15 species were found only in our studies).

### 2.3. Molecular Analysis

#### 2.3.1. Phylogenetic Analysis

**Figure 4:**
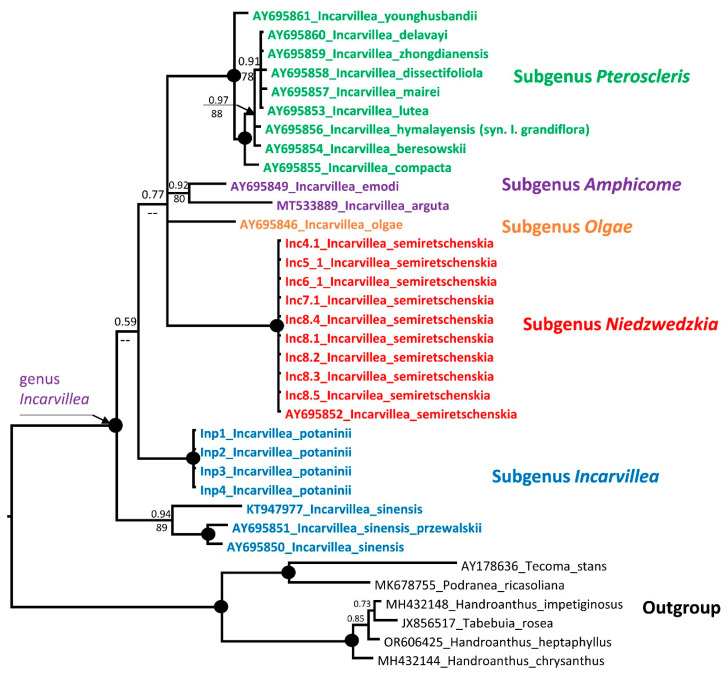
Phylogenetic nrITS tree of the genus *Incarvillea*. The joint presence of Bayesian with a probability greater than 0.98 and bootstrap support greater than 95% is indicated by a black dot. If the bootstrap values were below 50% they are displayed with --. The accession number of sequences from the NCBI GenBank is given with the species name. For the origin of Inc and Inp accessions, see Table 2.

nrITS analysis: We sequenced ITS with 10 accessions of *I. semiretschenskia* and 4 accessions of *I. potaninii*. All sequences within a species are identical. The ITS alignment of 35 accessions, including 16 *Incarvillea* sequences from GenBank and 6 accessions as the outgroup, is 605 bp long (see Supplementary File S1A). In these sequences, 443 characters were constant, 44 variable characters were parsimony-uninformative, and 118 were parsimony-informative. Unweighted parsimony analysis resulted in one most parsimonious tree of 308 steps (CI = 0.6883; RI = 0.8879). The substitution model HKY + G was chosen according to the AIC in jModelTest2 v.2.1.6 for the Bayesian analysis (Figure 4). *Incarvillea* is monophyletic, and the *I. sinensis* accessions (subgen. *Incarvillea*) form a basal clade in the genus with medium support (0.94 PP and 89 BS). *I. potaninii* accessions (subgen. *Incarvillea*) also form a clear monophyletic clade and the sister clade to *I. sinensis*, leaving the subgenus *Incarvillea* as weakly monophyletic. All remaining subgenera form unresolved clades with monophyletic clades with varying levels of support: subgenera *Pteroscleris* and *Niedzwedzkia* have very strong support, and subgenera *Amphicome* and *Olgae* are weakly supported or unresolved. This may be due to only one accession sequence existing for one species. Interestingly, the ITS tree topology differs in Bayesian, maximum likelihood, and maximum parsimony. In the maximum likelihood and maximum parsimony trees, *I. semiretschenskia* is sister to all other *Incarvillea* species, and the subgenus *Incarvillea* is more clearly paraphyletic than in the Bayesian tree. It should also be noted that support for separating the clade containing *I. semiretschenskia* from other species of the genus in the maximum likelihood tree is minimal (51%) (Figure S1).

Plastid analysis: We sequenced two plastid fragments: trnL-trnF (including the trnLUAA intron, trnLUAA exon, and trnF spacer) and the trnH-psbA intergenic spacer (IGS) in *I. semiretschenskia* and *I. potaninii*. Unfortunately, the trnH-psbA IGS sequences are not available for all Incarvillea species, which limits the scope of our analysis. Therefore, we do not present a combined plastid tree here, highlighting the need for further research in this area.

**Figure 5:**
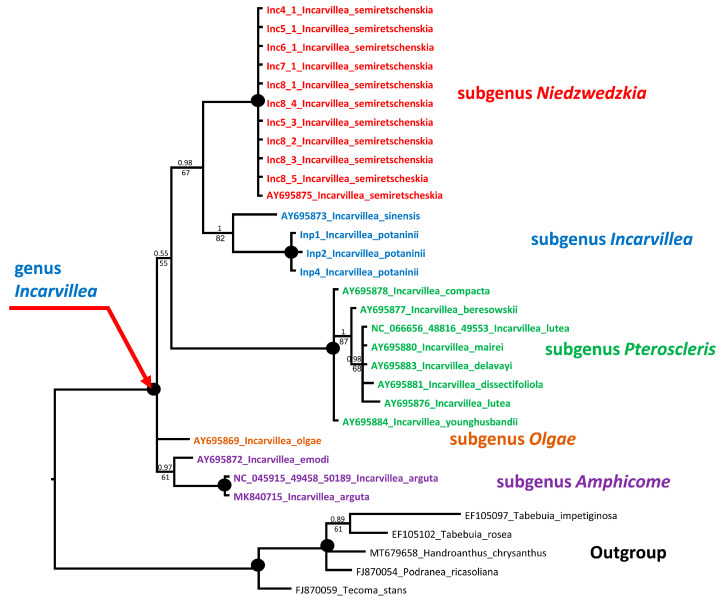
Phylogenetic plastid tree, based on trnL-trnF sequences of the genus *Incarvillea*. The joint presence of Bayesian with a probability greater than 0.98 and bootstrap support greater than 95% is indicated by a black dot. The accession number of sequences from the NCBI GenBank is given with the species name. For the origin of Inc and Inp accessions, see Table 2.

We sequenced the plastid trnL-trnF fragment with 10 accessions of *I. semiretschenskia* and 3 accessions of *I. potaninii*. All sequences within a species are identical. The alignment of 32 accessions of trnL-trnF sequences, including 13 *Incarvillea* sequences from GenBank and 5 accessions as an outgroup, is 815 bp long (see Supplementary File S1B). In these sequences, 691 characters were constant, 39 variable characters were parsimony-uninformative, and 85 were parsimony-informative. Unweighted parsimony analysis resulted in nine most parsimonious trees of 149 steps (CI = 0.8993; RI = 0.9673). The substitution model GTR + G was chosen according to the AIC in jModelTest2 v.2.1.6 for the Bayesian analysis (Figure 5). The genus *Incarvillea* is clearly monophyletic with very strong support. All subgenera are also monophyletic, whereby the subgenera Olgae and Amphicome form unresolved basal clades but with very weak support (0.58 PP and 67% BS). All other subgenera form the sister clade to them. Subgenus *Niedzwedzkia* forms the sister clade to subgenus *Incarvillea*.

**Figure 6:**
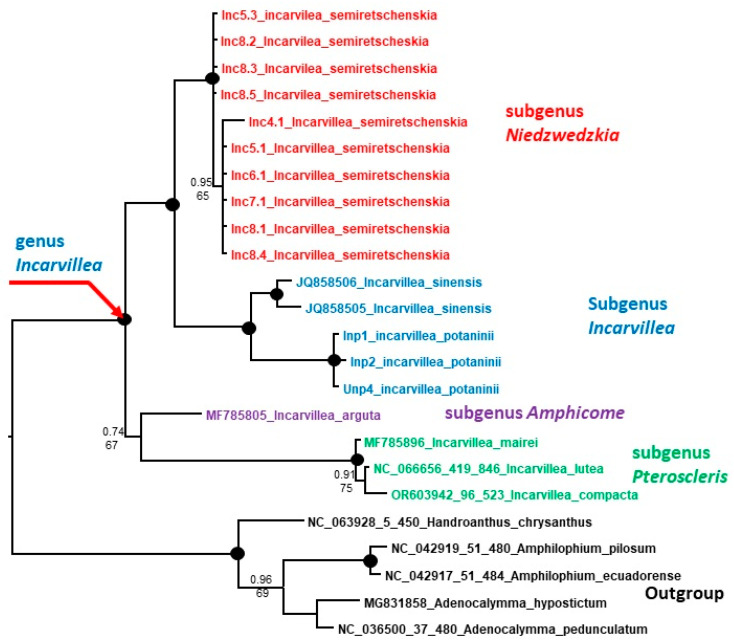
Phylogenetic plastid tree, based on trnH-psbA IGS sequences of the genus *Incarvillea*. The joint presence of Bayesian with a probability greater than 0.98 and bootstrap support greater than 95% is indicated by a black dot. The accession number of sequences from the NCBI GenBank is given for species name. For the origin of Inc and Inp accessions, see Table 2.

We sequenced the plastid trnH-psbA IGS with 10 accessions of *I. semiretschenskia* and 3 accessions of *I. potaninii*. All sequences within the accessions of *I. potaninii* are identical. However, the sequences of *I. semiretschenskia* have some single adenine versus cytosine exchanges. This affects three accessions from population 8 and one accession from population 5. The alignment of 24 accessions of trnH-psbA sequences, including 6 *Incarvillea* sequences from GenBank and 5 accessions as an outgroup, is 475 bp long (see Supplementary File S1B). In these sequences, 342 characters were constant, 28 variable characters were parsimony-uninformative and 105 were parsimony-informative. Unweighted parsimony analysis resulted in one of the most parsimonious trees of 170 steps (CI = 0.8824; RI = 0.9482). The substitution model GTR + G was chosen according to the AIC in jModelTest2 v.2.1.6 for the Bayesian analysis (Figure 6). The topology of the trnH-psbA tree (Figure 6) is slightly different from that of the trnL-trnF tree (Figure 5). Here, subgenera *Pteroscleris* and *Amphicone* are basal to the clade containing subgenera *Incarvillea* and *Niedzwedzkia*. This can be explained by missing sequences from subgenus *Olgae* and other *Incarvillea* species.

#### 2.3.2. Divergence Time Estimation

**Figure 7:**
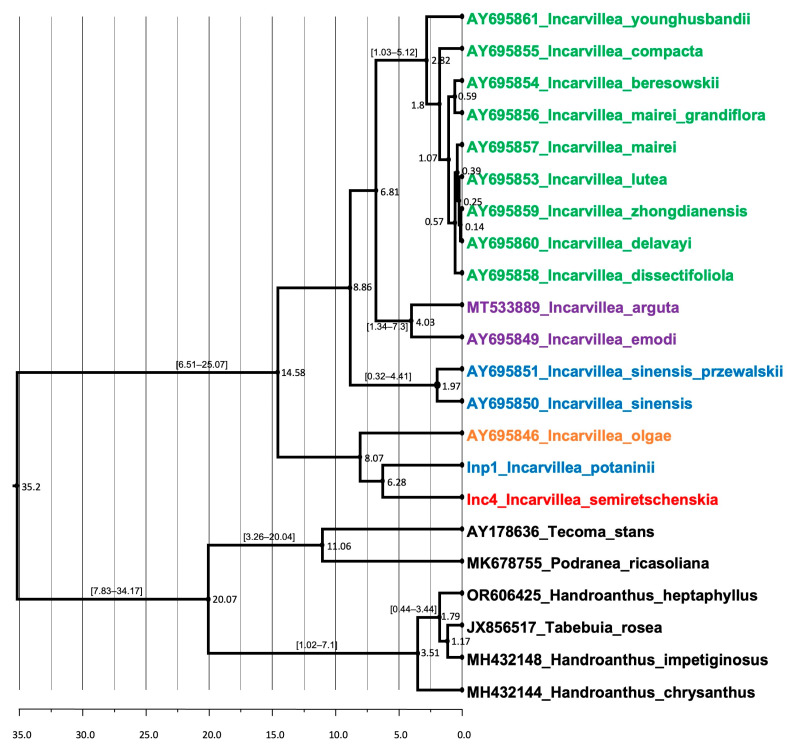
Dated phylogeny based on ITS sequences of the genus *Incarvillea*. The median rate is given in units of substitutions per million (95% HPD) above the nodes, and the nodes’ numbers represent the absolute ages in million years.

Our divergence time estimation analyses of the genus *Incarvillea* (Figure 7) generated a slightly different to the nrITS topology, compared to the Bayesian inference analysis (Figure 4), but corresponded to the maximum likelihood tree (Figure S1). According to the analyzed taxon sets, consisting of only one accession for each taxon, the genus *Incarvillea* underwent significant diversification. It diversified from the genera in the tribe Tecomeae of the Bignoniaceae family around 35.7 million years ago (mya) in the middle Eocene. The first diversification within the genus *Incarvillea* into two sister clades, A, which includes only three north-distributed *Incarvillea* species—*I. semiretschenskia*, *I. potaninii,* and *I. olgae*—and B, which includes all other species of the genus *Incarvillea*, occurred only in the middle Miocene (14.58 Mya), providing a clear and engaging timeline of the genus’s evolution. *I. semiretschenskia* and *I. potaninii* are sister taxa that diverged in the late Miocene (6.28 Mya).

#### 2.3.3. Fingerprint Analysis

ISSR-PCR and SCoT markers were used to study the intraspecific variability of *I. semiretschenskia*. All 13 samples from both populations in Shilozek (A) and Tyrnakty (B) sites were included in the fingerprint analysis. As a result of using five ISSR-PCR and seven SCoT markers (Table 2), 36 polymorphic ISSR fragments from 50 analyzed amplicons were identified. A total of 65 polymorphic SCoT fragments from 81 analyzed amplicons were identified on 13 samples depending on the primer; 8 to 16 amplified DNA fragments (bands) were detected, and the sizes of the fragments varied from 500 to 3200 bp. A separate UPGMA analysis of the ISSR and SCoT matrices is seen in Figure S2. In the main analysis, we combined both matrices. Analysis of the ISSR and SCoT spectrum of the natural population of *I. semiretschenskia* revealed 131 amplified sections of DNA, of which 101 were polymorphic. On average, the ISSR and SCoT loci polymorphism level identified using 12 primers was 77.1% (Table 3).

**Figure 8:**
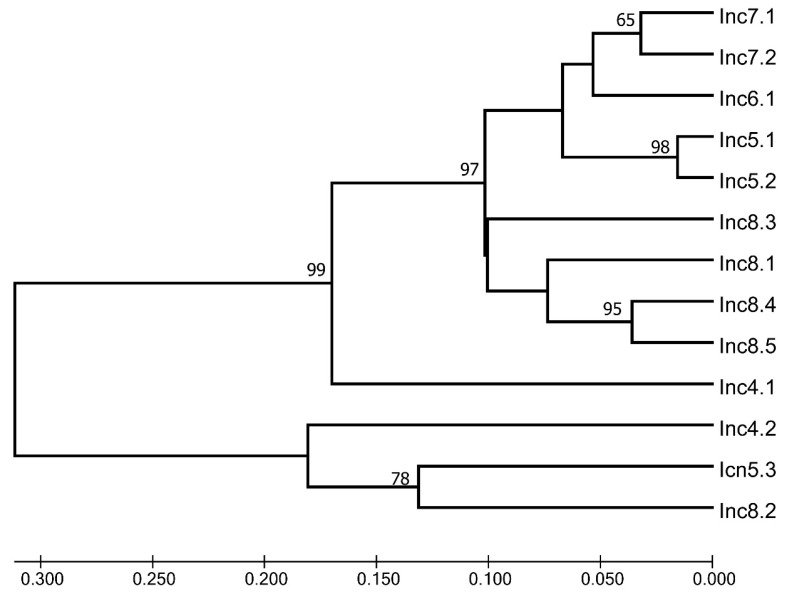
UPGMA tree based on combined ISSR and SCoT matrixes. See Table 3 and Figure 9 for the origin of Inc accessions.

The results indicate the studied samples’ high molecular genetic polymorphism (77.1%) and confirm the promising prospects of using fingerprint markers to establish genetic differences within *I. semiretschenskia*. In this study, a dendrogram constructed using an unweighted pair-group method using arithmetic means (UPGMA) showed the separation of all the studied samples into two clusters (Figure 8).

One cluster consists of three accessions from populations A (Inc4.2 and Inc5.3) and B (Inc8.2), and the other cluster combines all remaining accessions examined, with accession Inc4.1 standing somewhat isolated. The PCA of the same fingerprint matrix in the two first analyses (component 1 with component 2 and component 1 with component 3: both constellations explain over 50% of variability) (Figure S3) shows similar results.

## 3. Discussion

### 3.1. Ecological Conditions

The ecological conditions of *I. semiretschenskia* habitats are rocky slopes and intermountain valleys of the low mountains with light chestnut mountain soils with little development distributed in the steppe altitudinal belt. In these habitats, the dominating or co-dominating species are typical of light chestnut mountain soils with a shortened profile and poorly expressed genetic horizons, containing insignificant amounts of organic matter with low nutrient availability and having a light granulometric composition determining surface passivity in terms of absorption capacity. Single individuals are found in the conditions of intermountain elevated valleys with the development of light chestnut, incompletely developed mountain soils characterized by a profile of greater thickness with a certain set of genetic horizons, including humus-accumulative, illuvial, or illuvial-carbonate horizons. The sparse distribution of *I. semiretschenskia* is caused by the relatively young age of secondary relief and soils of the elevated valleys in relation to the conditions of rocky slopes and peaks of low mountains. A significant role in the distribution of the species is determined by the predominant development of the dwarf shrub *H. songaricum* in the vegetation cover. In conditions of anthropogenic disturbance associated with the development and extraction of limestone, a fragmentary increase in the abundance of *I. semiretschenskia* population is noted, which is caused by some improvement in aeration during the destruction of rocks, loosening the substrate, which creates conditions for the recovery of the population.

### 3.2. Floristic Diversity

Analyzing a list of flora of *I. semiretschenskia* communities (Table S1), we came to the conclusion that the spectrum of the leading families (Amaranthaceae, Asteraceae, Brassicaceae, Rosaceae, Fabaceae, and Poaceae) is typical for the deserts of Central Asia [24,29]. The spectrum of the leading genera demonstrates its proximity to the desert steppes and ecosystems of xeropetrophytic ephemeroid–bunch grass–dwarf semishrub, ephemeroid–sagebrush–dwarf semishrub, and grass–shrub–dwarf semishrub desert steppes on mountainous, light chestnut, underdeveloped soils of low mountains [17], in which, along with bunch grasses and feather grass, the presence of sagebrush species and other semishrubs is widespread. In total, five endemics (*Jurinea robusta* Schrenk, *Haplophyllum multicaule* Vved., *I. semiretschenskia*, *Phlomoides septentrionalis*, and *Schrenkia involucrata*) and five species listed in the Red Data Book of Kazakhstan (*Jurinea robusta*, *I. semiretschenskia*, *Iris kuschakewiczii*, *Tulipa alberti* Regel, and *T. biflora*) [3] are distributed in the habitats of *I. semiretschenskia*.

### 3.3. The Influence of Anthropogenic Factors

The anthropogenic impact was indicated by Baitulin and Sinitsyna [7] and Kokoreva et al. [5,8]. Grazing and fires were identified as the main factors. As a result of the fires, populations were completely burned out and did not recover over the 6 years of observation. The influence of grazing leads to a decrease in the species composition and an increase in the abundance of weeds (*Alyssum desertorum*, *Capsella bursa-pastoris* (L.) Medik., *Centaurea virgata* subsp. *squarrosa*, and *Cousinia affinis*, *Lappula microcarpa)*. Winterholler [1] notes that sheep eat fresh shoots in early spring and do not touch flowering plants but eat mature capsules in autumn.

Our research has shown that the habitats of *I. semiretschenskia* are disturbed to different degrees. There is grazing on the Turnakty site (B), where the degree of disturbance is average, while the Shilozek site (A) is severely disturbed and the leading factor is technogenic. There are 18 weed species growing in the studied areas, but none was found in large abundance. The most common include *Alyssum desertorum*, *Bromus tectorum*, *Cousinia affinis*, *Eremopyrum orientale*, *Lappula macrocarpa,* and *Poa bulbosa*. Medium grazing influenced *Centaurea virgata* subsp. *squarrosa*, *Eragrostis minor*, *Meniocus linifolius*, *Ranunculus testiculatus*, *Secale sylvestre*, and *Strigosella africana*. In total, 27.5% of weed species were found in technogenic areas, including *Bothriochloa ischaemum*, *Brassica elongata* ssp. *integrifolia Ceratocarpus arenarius,* and *Marrubium anisodon*. In natural areas under the influence of grazing, 20% of the species are weeds. It should be noted that no alien species were found in the studied habitats. This indicates that apophytes, the native plants of ruderal nature, increase in number and abundance when disturbed.

The analysis of the floristic composition of the two sites showed that anthropogenic disturbances led to a significant reduction in the species composition of communities, the loss of rare and endemic species, and a decrease in the projective coverage of *I. semiretschenskia.* The activities of the limestone quarry have destroyed most of the limestone ridge with populations. With the further continuation of mining operations, there is a danger of completely losing communities with the loss of habitats, as highlighted also for the communities of other rare plant species [31].

### 3.4. DNA Molecular Analysis

#### 3.4.1. Phylogenetic Analysis

Our phylogenetic analysis confirms the monophyly of the genus *Incarvillea*, and *I. semiretschenskia* (*Niedzwedzkia*) undoubtedly belongs to the genus *Incarvillea*. However, the position and relationship of *I. semiretschenskia* within the genus *Incarvillea* is not very clear. All subgenera in the genus *Incarvillea* are monophyletic solids, with only one exception: the subgenus *Incarvillea* with *I. sinensis* and *I. potaninii* form two separate clades with weak support in the ITS tree (Figure 4). Furthermore, the separation of the well-supported monophyletic clades of subgenera is very weakly supported in all phylogenetic trees (Figure 4, Figure 5 and Figure 6), which leads us to suspect that the differentiation of the subgenera in the genus *Incarvillea* happened very abruptly and probably simultaneously. The fact that the phylogenetic signal of nrITS is not very clear is shown by the different positions of *I. semiretschenskia* in the topology of the Bayesian and maximum likelihood trees (Figure 4 and Figure S2).

#### 3.4.2. Dated Phylogeny of the Genus *Incarvillea*

Our estimated dating of diversification in the genus *Incarvillea* is slightly younger (14.58 mya) than estimated by Rana et al. [21], which may be explained by the inclusion of *I. potaninii* in our analysis or the different estimation procedures used by Rana et al. [21,32]. Nevertheless, the differences are not so dramatic; even the time of differentiation within the genus *Incarvillea* is evident in all works on the middle and later Miocene. This is when the strong increase in the Qinghai–Tibetan Plateau’s (QTP) impact on aridization north of the QTP emerged [33].

#### 3.4.3. Fingerprints Analysis

The fingerprint analysis results show a large genetic variability in the *I. semiretschenskia* population studied (77,1% average level of polymorphism) and that the populations are not isolated because the plants of populations A and B are not monophyletic. Some outliers (accessions Inc4.2, Inc5.3 from population A, and Inc8.2 from population B) form their own clade (see Figure 7 and Figure S3). Nevertheless, we consider the fingerprint analysis preliminary and believe that the analysis should be extended to all possible known populations and with a much larger number of plants from each population. Additionally, more modern methods can be used; for example, next-generation sequencing, like genotyping by sequencing (GBS) or similar approaches.

### 3.5. In Situ and Ex Situ Conservation

The continued existence of the rare species *I. semiretschenskia* depends on our attitude to its preservation in nature and culture. The main task in the near future should be to identify all existing populations in nature and organize protected areas within their borders. B.A. Winterholler and I.O. Baitulin have repeatedly discussed the protection of this rare relic species. The number of individuals in *I. semiretschenskia* populations was estimated to be from 25,000–28,000 [34] to 30,000–32,000 [35]. According to more recent data by Kokoreva et al. [8], the number of individuals in the area of 10 hectares was 31,000. Our studies have shown that the population density has now decreased, which tended to decrease the number of studied populations. We understand that, so far, there is no reason to classify *I. semirechenskia* as a “Plant species with extremely small populations” (PSESP) with fewer than 5000 mature individuals in the wild and fewer than 500 in each isolated population [36]. However, a population that grows in a limestone quarry may become a PSESP in the near future without our conservation efforts. Reintroduction measures should be carried out in such disturbed habitats. Our observations have revealed that there is potential for population recovery even in severely disturbed anthropogenic habitats by sowing seeds in crushed limestone in micro-niches where possible.

The growth of *I. semiretschenskia* in botanical gardens has a rich history. The foundation was laid in 1937 by Rusanov in the Botanical Garden of the Central Asian State University [2,37,38].

In the Botanical Garden of Almaty City, seed sowing was carried out for the first time first in laboratory conditions, and then on experimental plots from seeds from the Suvorov expedition of 1952 [39]. Further experiments on introduction were continued [40,41,42], and the species was recommended for use in urban landscaping [9,43,44]. Later, a successful introduction was carried out in the conditions of Central Kazakhstan (the cities of Karaganda and Zhezkazgan) [45,46,47,48]. In Zhezkazgan City (Ulytau region), this species has been tested not only in the botanical garden, where it is stable in culture and self-seeding, but also in urban landscaping, which was the first experiment of its kind. Moreover, the climatic conditions of Central Kazakhstan are more severe compared to the place of origin of the species (the Shu-Ile Mountains). According to the Zhezkazgan meteorological station, the average annual precipitation is 208 mm, the average annual air temperature is +4.3 °C, the average monthly temperature in January is −21 °C (the absolute minimum is −50 °C), the average monthly temperature in July is +28.2 °C (the absolute maximum is +43 °C), and the sum of air temperatures above 10° is 3013° [13]. Good results from the introduction in botanical gardens of different regions of Kazakhstan allow us to hope that the species has the potential to survive in conditions of climate change.

*I. semiretschenskia* has also been successfully tested in the Botanical Gardens of Kyrgyzstan [49] and Ukraine [50,51].

At present, this unique relic plant grows in the living collections of the Main Botanical Garden (Almaty City) and the Botanical Garden of Zhezkazgan City [52,53]. Seeds are stored in the seed bank of the Institute of Botany and Phytointroduction [54].

## 4. Materials and Methods

### 4.1. Data Collection

**Figure 9:**
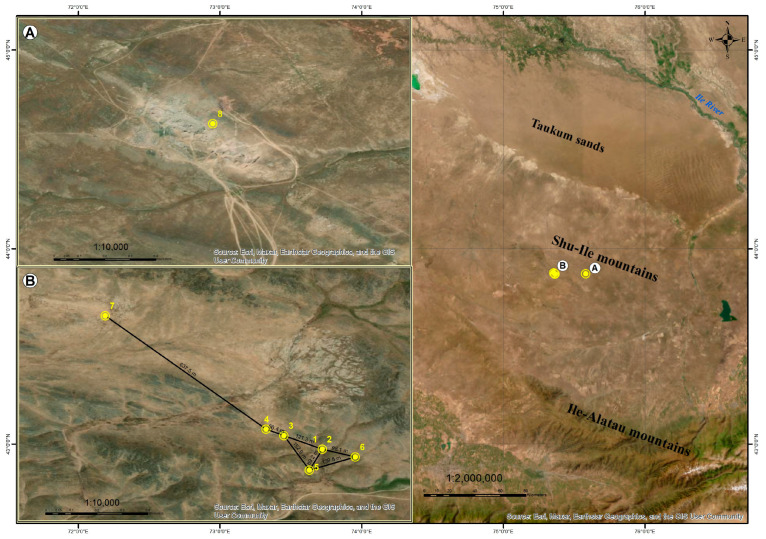
Location of the studied populations. Shilozek site (**A**). Tyrnakty site (**B**). *I. semiretschenskia* populations (1–8).

The studies were conducted on 16–19 May 2023, in two low mountain massifs: Shilozek (A) (812 m asl.) and Tyrnakty (B) (1063–1075 m asl.) (Figure 9). Both arrays differ in their degree of anthropogenic impact. Massif A (Shilozek site) turned out to be in a limestone quarry, where the environment is very severely disturbed. Massif B (Tarnakty site) is in a natural habitat, the degree of disturbance is average, and the anthropogenic factor is grazing.

Seven populations were surveyed at site B. These populations are located at distances ranging from 56 to 638 m of each other (in total, 913 m from the extreme points). Since the populations are located nearby, it was decided to accept them as one population, which was divided into plots. The area of the *I. semiretschenskia* population within the boundaries of the plant community and the plant density per 100 sq m were determined at each site, and samples were selected for genotyping.

The vegetation was studied using the traditional methods of geobotanic field research [55,56]. The studied sites were 10 × 10 sq m. The geolocation was registered using a Global Positioning System (GPS) device, and detailed geobotanical descriptions were compiled. The coordinates, landscapes, soils, water regime, total projective coverage, layers, and degree of transformation were defined for each plant community. The full floristic composition was given, and the phenological phases of the plant species, vigor (according to a five-point scale), spread (using Bykov’s scale), morphometric parameters (height, habitus), and abundance (using the Drude scale) [57] were defined. Symbols of Drude’s scale indicate the frequency of occurrence/coverage of a species. The symbols are as follows: soc (socialis)—the dominant species, frequency of occurrence/coverage exceeds 90%; cop^3^ (copiosus)—an abundant species, frequency of occurrence/coverage is up to 80%; cop^2^—a species is represented by numerous individuals, frequency of occurrence/coverage is up to 20%; cop^1^—frequency of occurrence/coverage is up to 4%; sp (sparsus)—frequency of occurrence/coverage about 0.8%; sol (solitarus)—scanty individuals, frequency of occurrence/coverage not exceeding 0.16%; and un (unicum)—a single individual. Descriptions of the vegetation were produced using the vegetation description forms.

The identification of the plant species was carried out on the basis of the identification keys of the nine-volume Flora of Kazakhstan [58,59,60,61,62,63,64,65,66] and the two-volume Illustrated Guide for Identification of the Plants of Kazakhstan [67,68]. The names of the plant species, genera, and families were quoted in accordance with the online resource Plants of the World Online [69]. To determine the similarity of the species composition of the two populations of *Incarvillea*, the Sörensen coefficient was used [70].

### 4.2. Soil Analysis

The research was carried out according to standard methods generally accepted in soil science in field and laboratory conditions [71,72]. In the field, two soil profiles were laid in natural habitats, from which samples were taken for chemical analysis.

Chemical analyses were carried out in the laboratory of the U.U. Uspanov Kazakh Research Institute of Soil Science and Agrochemistry in Almaty. Soil sampling, storage, and laboratory experiments were conducted in accordance with the Interstate Standard GOST:17.4.3.01-83 [73], measurement of calcium and magnesium content in soils was carried out in accordance with the Interstate Standard GOST 26428-85 [74], pH content was determined according to GOST 26423-85 [75], and humus content was measured according to ST RK 34477-2019 [76] and GOST.26213-91 [77]. Tyurin and granulometric composition of soil were determined in accordance with state standard GOST 12536-2014 [78], and water extraction was determined according to GOST 26423-85-26428-85 [79].

### 4.3. Taxon Sampling and DNA Sequencing

#### 4.3.1. Taxon Sampling

Silica-dried leaf material was collected from 13 plants of *I. semiretschenskia* in the field in the late spring of 2023, and four samples of *I. potaninii* from different areas in Mongolia were collected from Herbarium UBU (Table 3).

#### 4.3.2. DNA Isolation, Amplification and Sequencing

Total genomic DNA was isolated from leaves in silica gel using the InnuPREPP Plant DNA Kit (Analytic Jena AG) according to the manufacturer’s instructions and used directly in PCR amplification. We sequenced the internal transcribed spacer (ITS) from the nuclear ribosomal DNA of ten samples of *I. semiretschenskia* and four samples of *I. potaninii*. Additionally, the plastid trnL-trnF and trnH-psbA IGS were sequenced. For GenBank accession numbers of the sequenced regions, see Table 3. For most samples, the nrDNA ITS region (ITS1, 5.8S, and ITS2) was amplified using primers ITS-A [80] and ITS-4 [81]. Primers for the chloroplast regions were as described in Shaw et al. [82] for the trnH-psbA region and as described in Taberlet et al. [83] for trnL-trnF. To perform the PCR, the amplification protocol for Red Mix was used, involving a 20 µL reaction mixture with 2× HS Taq Mix Red (Biozym Scientific GmbH, Hessisch Oldendorf, Germany). The mix was 1 µL each of direct primer and reverse primer, 10 µL Red Mix, and 8 µL distilled water. The amplified products were tested via electrophoresis on a 1.5% agarose gel stained with ethidium bromide. The DNA fragments were visualized under UV light on a Gel I X20 Lmager (INTAS Science Imaging Instruments GmbH, Göttingen, Germany) and documented using a Mitsubishi P93D printer (Mitsubishi Elec. Corp., Chiyoda City, Japan). PCR products were sent to the Microsynth SeqLab [84]. The sequences of our samples were aligned, together with all known sequences of the genus *Incarvillea* from NCBI GenBank with CLUSTAL X [85], and corrected manually in MEGA 7 [86] where necessary.

#### 4.3.3. Phylogenetic Analysis

Several species from the tribe *Tecomeae* of the family Bignoniaceae that, according to the data of Rana et al. [21] and BLAST analysis, are closest to the genus *Incarvillea* were selected as an outgroup: *Tecoma stans* (L.) Juss. ex Kunth, *Podranea ricasoliana* (Tanfani) Sprague, *Handroanthus chrysanthus* (Jacq.) S.O.Grose, *Handroanthus heptaphyllus* (Vell.) Mattos, *Handroanthus impetiginosus* (Mart. ex DC.) Mattos, and *Tabebuia rosea* (Bertol.) DC. Both datasets (nrITS and the cpDNA markers) were analyzed separately through Fitch parsimony with the heuristic search option in PAUP version 4.0 b10 [87] with MULTREES, TBR branch swapping, and 100 replicates of random addition sequences. Gaps were treated as missing data. The most parsimonious trees according to the analysis were summarized in one consensus tree using the strict consensus method. Bootstrap support (BS) was performed using 1000 pseudoreplicates to assess the support of the clades [88]. Bayesian phylogenetic analyses were also performed using MrBayes 3.1.23 [89]. The sequence evolution model was chosen according to the Akaike information criterion (AIC) obtained from jModelTest2 [90]. Two independent analyses with four Markov chains were run for 10 million generations, sampling trees every 100 generations. The first 25% of the trees were discarded as burn-in. The remaining 150,000 trees were combined into a single dataset, and a majority-rule consensus tree and posterior probabilities (PPs) were obtained.

#### 4.3.4. Divergence Time Estimation

BEAST 1.8 [91] was used to estimate the divergence times in the genus *Incarvillea*. The BEAUti 1.8 interface was used to create input files for BEAST, and the XML files were manually adjusted where necessary.

A reduced subset of 22 accessions was selected from the ITS sequences for the divergence time estimation. We used the uncorrelated lognormal relaxed clock (ucld) with the substitution model selected according to the AIC (GTR + I + G). The Yule process was chosen as the speciation process. The ucld mean for the ITS data was set to a normal distribution, with a mean of 4.13 × 10^−9^ substitutions per site per year (sub/site/yr) and a standard deviation (SD) of 1.0 × 10^−9^, according to the works of Kay et al. [92]. The user-specified starting trees were inserted in the XML files in Newick format. Other parameters were set to default. Several short BEAST runs were first performed to examine the Markov chain Monte Carlo (MCMC) performance. Additional runs with empty alignments were carried out to ensure that the priors alone were not determining the results. Finally, three independent BEAST runs were performed for every substitution rate setting with the MCMC chain length of 108 generations and a sample frequency of every 100.

The effective sample size (ESS) values were >200 with a 25% burn-in for all parameters, as confirmed by analyzing the output files with TRACER. The tree output files from BEAST were summarized with LogCombiner 1.8 and annotated with the program TreeAnnotator 1.8, and the burn-in was set to 25% with the aid of TRACER. The mean node heights option was selected, and the posterior probability was set to 0.5. The trees were visualized using FigTree 1.4.0 with mean ages and 95% highest posterior density (HPD).

#### 4.3.5. Fingerprints Methods

Start codon targeting (SCoT) [93] and inter simple sequence repeat (ISSR) [94]: to assess genetic polymorphism inside the *I. semiretschenskia* population, samples were tested with 10 SCoT primers, and 7 primers showing polymorphism were selected for further analysis. Ten ISSR primers were also tested, and five primers showing polymorphism were selected. The PCR was performed in a Professional Thermocycler (Biometra, Göttingen, Germany), with the following program: pre-precipitation for 01:30 min at 94 °C, for 36 cycles (00:45 min—+94 °C, 00:45 min—+50 °C, 1:30 min—+72 °C), and the final step—6:00 min. at +72 °C and 90:00 min at 12 °C. The DNA was separated in an electrophoresis chamber using agarose gels with an agarose concentration of 1.5% in a TVE buffer using ethidium bromide. The duration of electrophoresis was 3.5–4 h at an electric field voltage of 85 V. The DNA was visualized using INTAS Science Imaging with Intas GDS software. A 100 bp-DNA EXTENDED ladder was used as a DNA standard. The electrophoresis results were analyzed using the gel’s presence (1) or absence (0) of bands, followed by matrix generation. IBM SPSS Statistics was used to carry out PCA of the data. A dendrogram showing the degree of similarity between the populations studied and the genetic distance was constructed using the MEGA7.0 software [86]. To do this, the numeric values 1 and 0 in the matrix were replaced by alphabetic values (1 to A and 0 to G), and the sample names were formatted in Fasta format. For the matrix of SCoT data, see Supplementary File S1.

The combined matrix of SCoT and ISSR data was also analyzed in the SPSS program (Version 28, https://www.ibm.com/products/spss-statistics, accessed on 20 December 2022). This program executed a principal component analysis (PCA) based on a correlation matrix of characters (Supplementary File S1) using the Pearson correlation coefficient. Dendrograms were constructed using the unweighted pair group method with arithmetic mean (UNGMA) using the Mega7.0 program [86]. To do this, the numerical values 1 and 0 in the matrix were replaced with alphabetic ones (1 for T and 0 for A) and the sample names were formatted in FASTA format.

## 5. Conclusions

Our research has supplemented the existing data on the molecular phylogeny of *I. semiretschenskia*, the age of this species, the current state of its habitats, and threats to the species’ existence with respect to the current conditions of natural management. Despite the relatively detailed research on the species, interest in it since its description in 1915, and experiments on its introduction to botanical gardens around the world, most research has been exploratory in nature. In addition, there remain many unresolved questions on its phylogeny, on the ecological niche of the species for further in situ and ex situ conservation, and on its reintroduction into disturbed habitats. We consider our main task to be the creation of special, protected nature territories for the preservation of this unique relic species, which has survived since the Miocene and exists to this day in the Shu-Ile low mountains. Establishing small reserves or plant micro-reserves may be the most appropriate approach to preserve this rare species in situ [95]. Ex situ collections ensure that the preserved germplasm is available for future in situ restoration efforts. Seed banks and botanical gardens are given an active role in conservation programs. Once habitats where a species can maintain viable populations have been identified, an action plan should be developed that includes careful management, which is necessary to protect both the habitat and the species. Future activity may include population enhancement, relocation, or habitat restoration, as well as the study of useful properties of the plant [95,96].

The legislative base in Kazakhstan allowing the conservation of rare species and plant communities is regulated by two laws: “On Specially Protected Natural Areas” [97], and a new law “On Plant World” [98]. Sustainable conservation of populations and communities implies the preservation of predominantly natural growing conditions of plants. In case of threats to individual plant species, their populations, communities, and places of growth, state bodies establish and define special regimes of protection in the areas of their growth to give them the status of the state natural reserve fund and specially protected natural areas.

Through the efforts of our colleagues Alexander Dubynin and Vladimir Epiktetov, *I. semiretschenskia* has most recently been assessed for inclusion in the International Union for Conservation of Nature’s Red List of Threatened Species in 2024. *I. semiretschenskia* is listed as Endangered under criteria B1ab(iii)+2ab(iii) [99,100]. This confirms that the species is at high risk of global extinction. Therefore, further activity should be aimed at preserving the species in nature and organizing a natural reserve in the habitats of its populations.

## Figures and Tables

**Table 1:** Floristic composition of *I. semiretschenskia* habitats (TPC—total projective coverage, symbols of Drude’s scale, see Section 4.2).

Species	Abundance by Drude Scale	
	Tyrnakty (B)	Shilozek (A)
	1063–1075 m asl.	812 m asl.
	TPC: 35–40%	TPC: 25–30%
Shrubs and dwarf shrubs		
*Atraphaxis compacta* Ledeb.	sol	-
*A. pyrifolia* Bunge	sol	-
*A. spinosa* L.	-	sol
*Ephedra intermedia* Schrenk & C.A.Mey.	sol	un-sol
*Helianthemum songaricum* Schrenk ex Fisch. & C.A.Mey.	sp	-
*Prunus griffithii* var. *tianshanica* (Pojark.) Ingram	sol	sol
*Spiraea hypericifolia* (Pojark.) Ingram	sp	sp
Semishrubs and dwarf semishrubs		
*Artemisia juncea* Kar. & Kir.	sol	sp
*A. sublessingiana* (B.Keller) Krasch. ex Poljakov	sol	sol
*Bassia prostrata* (L.) Beck	sol	sol
*Incarvillea semiretschenskia* (B.Fedtsch.) Grierson	sp-cop_1_	sp
*Krascheninnikovia ceratoides* (L.) Gueldenst.	-	sol
Perennial herbs		
*Artemisia turanica* Krasch.	sol-sp	-
*Allium petraeum* Kar. & Kir.	sol	sol-sp
*A. trachyscordum* Vved.	sol	-
*Astragalus chaetodon* Bunge	sol	sol
*A. schrenkianus* Fisch. & C.A. Mey.	sol	-
*A. sieversianus* Pall.	sol	-
*Biebersteinia multifida* DC.	sol	-
*Bothriochloa ischaemum* (L.) Keng	-	un-sol
*Brassica elongata* subsp. *integrifolia* (Boiss.) Breistr.	-	un-sol
*Carex pachystylis* J.Gay	sp-sol	-
*Centaurea virgata* subsp. *squarrosa* (Boiss.) Gugler	sol	-
*Cousinia affinis* Schrenk ex Fisch. & C.A. Mey.	sol	sol
*Eremurus cristatus* Vved.	sol	sol
*Euphorbia rapulum* Kar. & Kir.	sol	sol
*Festuca valesiaca* Schleich. ex Gaudin	sp	sp
*Ferula ovina* Boiss.	un-sol	-
*F. tschuiliensis* Bajtenov	sol	-
*Gelasia circumflexa* (Krasch. & Lipsch.) Zaika, Sukhor. & N. Kilian	sol	sol
*Gentiana olivieri* Griseb.	sol	-
*Goniolimon cuspidatum* Gamajun.	sol	-
*G. speciosum* (L.) Boiss.	sol	-
*Haplophyllum acutifolium* (DC.) G. Don	sol	sol
*H. latifolium* Kar. & Kir.	sol-sp	-
*Iris albomarginata* R.C. Foster	sol	-
*I. halophila* var. *sogdiana* (Bunge) Skeels	sol	-
*I. kuschakewiczii* B. Fedtsch.	sol-sp	sol
*I. songarica* Schrenk ex Fisch. & C.A. Mey.	-	sol
*Ixiolirion tataricum* (Pall.) Schult. & Schult. f.	sol	-
*Jurinea adenocarpa* Schrenk ex Fisch. & C.A. Mey.	sol	sol
*Lagochilus platycalyx* Schrenk ex Fisch. & C.A. Mey.	sol	sol
*Linum perenne* L.	sol	-
*Marrubium anisodon* K. Koch	-	un-sol
*Oedibasis apiculata* (Kar. & Kir.) Koso-Pol.	un-sol	-
*Phlomoides molucelloides* (Bunge) Salmaki	un-sol	-
*P. septentrionalis* (Popov) Adylov, Kamelin & Makhm.	-	sol
*Piptatherum songaricum* (Trin. & Rupr.) Roshev.	sol-sp	-
*Poa bulbosa* L.	sp	sp
*Potentilla multicaulis* Bunge	sol	-
*P. soongorica* Bunge	-	sol
*Prangos cachroides* (Schrenk) Pimenov & V.N.Tikhom.	sol	-
*Pseudosedum affine* (Schrenk) A. Berger	sol	-
*Ranunculus platyspermus* Fisch. ex DC.	sol	-
*Rindera tetraspis* Pall.	sol	sol
*Rosularia glabra* (Regel & C.Winkl.) A. Berger	sol	-
*Scutellaria titovii* Juz.	un-sol	-
*Schrenkia involucrata* Regel & Schmalh.	sol	-
*Seseli sessiliflorum* Schrenk	sol	sol
*Sibbaldianthe bifurca* (L.) Kurtto & T. Erikss.	sol	-
*Stipa capillata* L.	sp-cop_1_	-
*S. caucasica* Schmalh.	sol	sol
*S. conferta* Poir.	-	sol
*S. hohenackeriana* Trin. & Rupr.	-	sol
*S. kirghisorum* P.A. Smirn.	sol-sp	-
*S. lessingiana* Trin. & Rupr.	sp-cop_1_	-
*S. orientalis* Trin.	sp-cop_1_	sol
*S. richteriana* Kar. & Kir.	sol	-
*S. sareptana* A.K. Becker	sol	-
*Tulipa biflora* Pall.	sol	-
*Tragopogon marginifolius* Pavlov	sol	-
*T. ruber* S.G. Gmel.	sol	sol
*Ziziphora clinopodioides* Lam.	sol	un-sol
Annual herbs		
*Alyssum desertorum* Stapf	sol	sol
*Bromus tectorum* L.	sp	sp
*Ceratocarpus arenarius* L.	-	sol
*Eragrostis minor* Host	sol	-
*Eremopyrum orientale* (L.) Jaub. & Spach	sol	sol
*Lappula microcarpa* (Ledeb.) Gürke	sol	sol
*Meniocus linifolius* (Stephan ex Willd.) DC.	sol	-
*Minuartia meyeri* (Boiss.) Bornm.	sol	-
*Ranunculus testiculatus* Crantz	sol	-
*Secale sylvestre* Host	sol	-
*Strigosella africana* (L.) Botsch.	sol	-
*Ziziphora tenuior* L.	sol	-
Number of species	74	40

**Table 2:** Origin of *Incarvillea* accessions for DNA analysis and GenBank accessions number of sequences.

Code	Origin	Coordinates	Herbarium	ITS	trnL-trnF	psbA-trnH
*Inkarvillea semiretschskia*	Kazakhstan					
Inc4-1	Shu-Ile Mountains, Tyrnakty tract	43.872642 N, 75.361853 E	AA	PP864255	PP869653	PP869666
Inc4-2						
Inc5-1	Shu-Ile Mountains, Tyrnakty tract	43.871202 N, 75.363378 E	AA	PP864256	PP869654	PP869667
Inc5-2						
Inc5-3					PP869655	PP869668
Inc6-1	Shu-Ile Mountains, Tyrnakty tract	43.869667 N, 75.359 E	AA	PP864257	PP869656	PP869669
Inc7-1	Shu-Ile Mountains, Tyrnakty tract	43.876648 N, 75.35617 E	AA	PP864258	PP869657	PP869670
Inc7-2						
Inc8-1	Spurs of the Shu-Ile Mountains, Shilozek tract	43.87278 N, 75.581902 E	AA	PP864259	PP869658	PP869671
Inc8-2				PP864260	PP869659	PP869672
Inc8-3				PP864261	PP869660	PP869673
Inc8-4				PP864262	PP869661	PP869674
Inc8-5				PP864263	PP869662	PP869675
*Incarvillea potaninii*	Mongolia					
Inp1	Bayankhongor prov. Shinejinst sum, Jinst mountain	44.559183 N, 99.292440 E	UBA, 5 September 1980, Ch. Sanchir	PP864264	PP869663	PP869676
Inp2	Umnogobi prov. Bayandalai sum, Zuramtai mountai	43.488454 N, 103.773991 E	UBA, 5 September 1976, Ch. Sanchir, Ts. Tseplev	PP864265	PP869664	PP869677
Inp3	Gobi-Altai aimak, Khuren Khana mountain, Muu sar valley	44.409609 N, 97.346536 E	UBA, 7 September 1979, V.I.Grubov, A. Muldashaev	PP864266		
Inp4	Umnogobi prov. Takhilgyn khyr, 21 km south Noyon sum	43.117826 N, 102.132012 E	UBA, 8 September 1979, V.I.Grubov, A. Muldashaev	PP864267	PP869665	PP869678

**Table 3:** Fingerprints used primer and the number of amplified fragments.

Primer	Primer Sequence (5′-3′)	Number of Fragments	Polymorphic Fragments	Polymorphism
ISSR UBC841	(GA)8YC	11	9	81.8%
ISSR UBC884	HBH(AG)7	10	10	100%
ISSR HB12	(CAC)3 GC	10	5	50%
ISSR HB13	(GAG)3GC	11	8	72.7%
ISSR HB14	(CTC)3GC	8	4	50%
Ʃ ISSR		50	36	72%
SCoT 2	CAACAATGGCTACCACCC	11	9	81.8%
SCoT 7	CAACAATGGCTACCACGG	9	8	88.9%
SCoT 8	CAACAATGGCTACCACGT	12	11	91.7%
SCoT 12	ACGACATGGCGACCAACG	16	12	75%
SCoT 13	ACGACATGGCGACCATCG	8	7	87.5%
SCoT 14	ACGACATGGCGACCACGC	13	9	69.2%
SCoT 16	ACCATGGCTACCACCGAC	12	9	75%
Ʃ SCoT		81	65	80.2%
Average level of polymorphism				77.1%

## Data Availability

The datasets generated during the study are available from the corresponding authors upon reasonable request.

## Supplementary Materials

The following supporting information can be downloaded at: https://www.mdpi.com/article/10.3390/plants13233299/s1, Figure S1: Maximum likelihood tree of genus *Incarvillea*. Numbers above the nodes show the bootstrap support; Figure S2: UPGMA trees based on matrixes: A-ISSR; B—SCoT data. For the origin of Inc accessions, see Table 3; Figure S3: Interspecific location of 13 accessions of *Incarvillea semiretschenskia* in the PCA (SPSS): A—Components 1 and 2; B—Components 1 and 3; C—Components 2 and 3. For the origin of Inc accessions, see Table 3; Table S1: List of vascular plants of *Incarvillea semiretschenskia* communities in the Shu-Ile low mountains, File S1 (A,B) Alignments ITS and trnL-trnF.
