# Supplementary material for: Habitats, Plant Diversity, and Molecular Phylogeny of Endemic Relic Species Incarvillea semiretschenskia (Bignoniaceae)
Source: Plants (Basel). 2024 Nov 23;13(23):3299. doi: 10.3390/plants13233299 (PMC11644528; doi:10.3390/plants13233299)
Supplement: Supplementary file 1 [file plants-13-03299-s001.zip › Table S1. List of vascular plants.pdf]

Supplementary data

Table S.1. List of vascular plants of *Incarvillea semiretschenskia* communities in the Shu-Ile low mountains

Species / Sources of information	1	2	3	4	5	6	7	8	9
Amaranthaceae Juss.									
Anabasis cretacea Pall.	+	-	-	-	-	-	-	-	-
Bassia prostrata (L.) Beck	+	+	-	+	-	+	+	+	+
Caroxylon orientale (S.G.Gmel.) Tzvelev	-	+	-	-	-	-	-	-	-
Ceratocarpus arenarius L.	-	+	-	+	-	-	-	-	+
Girgensohnia oppositiflora (Pall.) Fenzl	-	-	-	+	-	-	-	-	-
Krascheninnikovia ceratoides (L.) Gueldenst.	-	+	-	-	+	-	-	-	+
Oreosalsola arbusculiformis (Drobow) Sennikov	-	-	+	+	-	-	-	-	-
O. laricifolia (Litv. ex Drobow)	+	+	-	-	-	-	-	-	-
Pyankovia brachiata (Pall.) Akhani & Roalson	-	+	-	-	-	-	-	-	-
Xylosalsola arbuscula (Pall.) Tzvelev	-	-	-	-	+	-	-	-	-
Amaryllidaceae J.St.-Hil.									
Allium caeruleum Pall.	+	-	-	-	-	+	-	-	-
A. kokanicum Regel	-	-	+	-	-	-	-	-	-
A. galanthum Kar. & Kir.	+	-	+	+		+	-	-	-
A. margaritae B.Fedtsch.	-	-	-	+	-	-	-	-	-
A. pallasii Murray	+	-	-	-	-	-	+	-	-
A. petraeum Kar. & Kir.	+	+	-	-	-	-	+	+	+
A. trachyscordum Vved.	-	-	-	-	-	-	+	+	+
A. vvedenskyanum Pavlov	-	-	+	-	-	-	-	-	-
Apiaceae Lindl.									
Eryngium planum L.	+	-	-	-	-	-	+	-	-
Ferula dissecta (Ledeb.) Ledeb.	-	-	+	+	-	-	-	+	-
F. karataviensis (Regel & Schmalh.)	-	-	-	-	-	-	+	-	-
F. ovina Boiss.	-	-	-	-	-	-	+	+	+
F. tschuiliensis Bajtenov	-	-	+	+	-	-	-	-	+
Hyalolaena bupleuroides (Schrenk) Pimenov & Kljuykov	-	-	-	+	-	-	-	-	-
Oedibasis apiculata (Kar. & Kir.) Koso-Pol.	-	-	-	-	+	-	-	+	+
Prangos cachroides (Schrenk) Pimenov & V.N.Tikhom.	-	-	-	-	-	-	-	+	+
Seseli glabratum Willd. ex Schult.	-	+	-	-	-	-	-	-	-
S. sessiliflorum Schrenk	-	-	+	+	-	-	-	+	+
* *Schrenkia involucrata* Regel & Schmalh.	+	-	-	+	-	-	+	-	+
Asphodelaceae Juss.									
Eremurus cristatus Vved.	+	-	-	+	-	-	+	+	+
Asteraceae Bercht. & J.Presl									

Arctium triflorum Kuntze	-	+	-	-	-	-	-	-	-
Artemisia heptapotamica Poljakov	-	-	-	+	-	-	-	+	-
A. juncea Kar. & Kir.	-	+	-	+	-	+	-	+	+
A. sublessingiana (B.Keller) Krasch. ex Poljakov	+	-	+	+	+	+	+	+	+
A. terrae-albae Krasch.	+	+	-	+	-	+	-	-	-
A. turanica Krasch.	-	-	-	-	-	-	-	+	+
Centaurea virgata subsp. squarrosa (Boiss.) Gugler	-	+	-	+	-	-	+	+	+
Cousinia affinis Schrenk ex Fisch. & C.A.Mey.	-	+	+	+	-	-	+	+	+
Gelasia circumflexa (Krasch. & Lipsch.) Zaika, Sukhor. & N.Kilian	-	-	-	+	-	-	-	-	+
Jurinea adenocarpa Schrenk ex Fisch. & C.A.Mey.	-	-	-	-	-	-	-	-	+
* + *J. robusta* Schrenk	-	-	-	-	-	+	-	-	-
Lactuca undulata Ledeb.	-	-	-	+	-	-	-	-	-
Scorzonera purpurea L.	-	-	+	-	-	-	-	-	-
Scorzoneroides autumnalis (L.) Moench	-	-	+	-	-	-	-	-	-
Takhtajaniantha pusilla (Pall.) Nazarova	-	+	+	-	-	-	-	-	-
Taraxacum sect. *Taraxacum* F.H.Wigg.	+	-	-	-	-	-	+	-	-
Tragopogon marginifolius Pavlov	-	-	-	-	-	-	-	-	+
T. ruber S.G.Gmel.	-	-	-	-	-	-	-	-	+
Biebersteiniaceae Schnizl.									
Biebersteinia multifida DC.	-	-	-	-	-	-	-	-	+
Bignoniaceae Juss.									
* + *Incarvillea semiretschenskia* (B.Fedtsch.) Grierson	+	+	+	+	+	+	+	+	+
Boraginaceae Juss.									
Lappula microcarpa (Ledeb.) Gürke	-	-	+	-	-	-	-	+	+
L. spinocarpos (Forssk.) Asch. ex Kuntze	-	-	+	-	-	-	-	-	-
Nonea caspica (Willd.) G.Don	-	-	+	-	-	-	-	-	-
Onosma dichroantha Boiss.	-	-	+	-	-	-	-	-	-
O. irritans Popov	-	+		+	-	-	-	-	-
Rindera tetraspis Pall.	-		+	-	-	-	-	-	+
Rochelia retorta (Pall.) Lipsky	-	-	-	+	-	-	-	-	-
Brassicaceae Burnett									
Alyssum alyssoides (L.) L.	-	-	+	-	-	-	-	+	-
A. dasycarpum Stephan ex Willd.	-	-	-	+	-	-	-	-	-
A. desertorum Stapf	+	-	+	+	-	+	-	-	+
Brassica elongata subsp. integrifolia (Boiss.) Breistr.	-	-	-	-	-	-	-	-	+
Erysimum czernjajevi N.Busch	-	-	-	+	-	-	-	-	-
E. diffusum Ehrh.	-	-	+	-	-	-	-	-	-
E. siliculosum (M.Bieb.) DC.	+	-	-	-	-	-	-	-	-

Goldbachia pendula Botsch.	-	-	-	+	-	-	-	-	-
Meniocus linifolius (Stephan ex Willd.) DC.	-	-	+	+	-	-	-	-	+
Strigosella africana (L.) Botsch.	-	-	+	+	-	-	-	-	+
Thlaspi arvense L.	-	-	+	-	-	-	-	-	-
Caprifoliaceae Juss.									
Valerianella cymbaecarpa C.A.Mey.	-	-	+	-	-	-	-	-	-
Caryophyllaceae Juss.									
Acanthophyllum pungens (Bunge) Boiss.	-	+	-	-	-	-	-	-	-
Dianthus crinitus subsp. *soongoricus* (Schischk.) Kozhevn.	-	-	-	+	-	-	-	-	-
D. kuschakewiczii Regel & Schmalh.	-	+	-	-	-	-	-	-	-
D. ramosissimus Pall. ex Poir.	-	-	-	-	-	-	+	-	-
Holosteum umbellatum subsp. *glutinosum* (M.Bieb.) Nyman	-	-	+	+	-	-	-	-	-
Minuartia meyeri (Boiss.) Bornm.	-	-	-	-	-	-	-	-	+
Silene gavrilovii (Krasn.) Popov	-	+	-	-	-	-	-	-	-
S. muslimii Pavlov	-	-	-	+	-	-	-	-	-
Cistaceae Juss.									
Helianthemum songaricum Schrenk ex Fisch. & C.A.Mey.	+	+	+	+	-	-	+	+	+
Crassulaceae J.St.-Hil.									
Pseudosedum affine (Schrenk) A.Berger	+	-	-	-	-	+	+	+	+
P. longidentatum Boriss.	-	-	-	+	-	-	-	-	-
Rosularia glabra (Regel & C.Winkl.) A.Berger	-	-	-	-	-	-	-	-	+
R. turkestanica (Regel & C.Winkl.) A.Berger	-	-	-	+	-	-	-	-	-
Cyperaceae Juss.									
Carex pachystylis J.Gay	-	-	-	-	+	-	-	+	+
Ephedraceae Dumort.									
Ephedra distachya L.	+	-	-	-	+	+	+	-	-
E. intermedia Schrenk & C.A.Mey.	-	-	-	-	-	-	-	+	+
Euphorbiaceae Juss.									
Euphorbia rapulum Kar. & Kir.	-	-	+	-	-	-	-	-	+
Fabaceae Lindl.									
Astragalus ammophilus Kar. & Kir.	-	-	+	-	-	-	-	-	-
A. chaetodon Bunge	-	-		+	-	-	-	-	+
A. filicaulis Fisch. & C.A.Mey. ex Ledeb.	-	-	+	-	-	-	-	-	-
A. kronenburgii B.Fedtsch. ex Kneuck.	-	-	+	+	-	-	-	-	-
A. pallasii Biehler	-	-	+	+	-	-	-	-	-
A. schrenkianus Fisch. & C.A.Mey.	-	-	-	-	-	-	+	-	+
A. sieversianus Pall.	-	-	-	-	-	-		+	+
Caragana aurantiaca Koehne	-	+	-	-	-	-	-	-	-

Medicago falcata L.	-	-	-	-	+	-	-	-	-
M. medicaginoides (Retz.) E.Small	+	-	-	+	-	-	-	-	-
Gentianaceae Juss.									
Gentiana olivieri Griseb.	-	-	-	-	-	-	+	+	+
Iridaceae Juss.									
Iris albomarginata R.C.Foster	-	-	-	-	-	-	-	+	+
+*I. kuschakewiczii* B.Fedtsch.	+	-	+	+	-	-	+	+	+
I. halophila var. *sogdiana* (Bunge) Skeels	-	-	-	-	-	-	-	-	+
I. songarica Schrenk ex Fisch. & C.A.Mey.	-	+	-	-	-	-	-	-	+
Ixioliriaceae Nakai									
Ixiolirion tataricum (Pall.) Schult. & Schult.f.	+	-	-	-	-	+	+	+	+
Lamiaceae Martinov									
Lagochilus hirtus Fisch. & C.A.Mey.	-	-	-	-	-	-	-	+	-
L. platycalyx Schrenk ex Fisch. & C.A.Mey.	-	+	-	+	-	-	-	+	+
Marrubium anisodon K.Koch	-	-	-	-	-	-	-	-	+
Phlomoides molucelloides (Bunge) Salmaki	-	-	-	-	-	-	+	+	+
* *P. septentrionalis* (Popov) Adylov, Kamelin & Makhm.	-	-	-	-	-	-	-	-	+
Scutellaria sieversii Bunge	-	-	-	+	-	-	-	-	-
S. titovii Juz.	-	+	-	-	-	-	+	-	+
Ziziphora clinopodioides Lam.	-	+	-	+	-	-	+	+	+
Z. tenuior L.	-	-	-	+	+	-	-	-	+
Liliaceae Juss.									
Gagea bulbifera (Pall.) Salisb.	+	-	-	-	-	+	-	-	-
+ *Tulipa alberti* Regel	-	-	-	+	-	-	-	-	-
+ *T. biflora* Pall.	+	-	-	+	-	+	+	+	+
Linaceae DC. ex Perleb									
Linum perenne L.	-	-	-	-	-	-	-	+	+
Plantaginaceae Juss.									
Linaria bungei Kuprian.	-	+	-	-	-	-	-	-	-
Veronica verna L.	-	-	-	+	-	-	-	-	-
Plumbaginaceae Juss.									
Goniolimon callicomum (C.A.Mey.) Boiss.	+	-	-	-	-	-	-	-	-
G. cuspidatum Gamajun.	-	+	-	+	-	-	+	+	+
G. speciosum (L.) Boiss.	-	-	-	+	-	-	-	-	+
Poaceae Barnhart									
Bothriochloa ischaemum (L.) Keng	-	+	-	-	-	-	-	-	+
Bromus japonicus subsp. *japonicus*	-	-	-	+	-	-	-	-	-
B. squarrosus L.	-	-	-	+	-	-	-	-	-
B. tectorum L.	-		+	+					+

Eremopyrum orientale (L.) Jaub. & Spach	+	-	+	+	-	+	-	-	+
Eragrostis minor Host	-	-	-	-	-	-	-	-	+
Festuca valesiaca Schleich. ex Gaudin	+	-	-	+	-	+	+	+	+
Piptatherum songaricum (Trin. & Rupr.) Roshev.	-	+	-	+	-	-	-	+	+
Poa bulbosa L.	+	+	+	+	+	+	+	+	+
P. bactriana subsp. *glabriflora* (Roshev.) Tzvelev	-	-	+	-	-	-	-	-	-
Secale sylvestre Host	-	-	-	-	-	-	-	-	+
Stipa capillata L.	+	+	-	-	-	+	+	+	+
S. caucasica Schmalh.	+	-	-	+	-	+	-	+	+
S. conferta Poir.	-	+	-	-	-	-	-	+	+
S. hohenackeriana Trin. & Rupr.	-	-	-	-	-	-	-	-	+
S. kirghisorum P.A.Smirn.	+	-	-	-	-	+	-	+	+
S. lessingiana Trin. & Rupr.	-	-	-	+	-	-	-	+	+
S. macroglossa P.A.Smirn.	-	-	-	+	-	-	-	-	-
S. orientalis Trin.	-	+	-	+	-	-	-	+	+
S. richteriana Kar. & Kir.	-	-	-	-	-	-	-	-	+
S. sareptana A.K.Becker	-	-	-	+	-	-	-	+	+
Polygonaceae Juss.									
Atraphaxis compacta Ledeb.	-	-	-	-	-	-	--	+	+
A. pyrifolia Bunge	+	-	-	-	-	-	+	+	+
A. spinosa L.	-	-	-	-	-	-	-	-	+
A. virgata (Regel) Krasn.	-	+	-	-	-	-	-	-	-
Polygonum polycnemoides Jaub. & Spach	-	-	-	+	-	-	-	-	-
Primulaceae Batsch ex Borkh.									
Androsace maxima L.	-	-	-	+	-	-	+	+	-
Ranunculaceae Juss.									
Ranunculus platyspermus Fisch. ex DC.	-	-	-	-	-	-	-	-	+
R. testiculatus Crantz	-	-	+	+	-	-	-	-	+
Rosaceae Juss.									
Potentilla multicaulis Bunge	-	-	-	-	-	-	-	-	+
P. soongorica Bunge	-	-	+	-	-	-	-	-	+
P. supina L.	-	-		-	-	-	+	-	-
Prunus erythrocarpa (Nevski) Gilli	-	+	-	-	-	-	-	-	-
P. griffithii var. *tianshanica* (Pojark.) Ingram	+	-	+	+	+	-	+	+	+
Rosa persica Michaut ex Juss.		-	+	-	-	-	-	-	-
Sibbaldianthe bifurca (L.) Kurtto & T.Erikss.	-	-	-	-	-	-	-	-	+
S. orientalis (Juz. ex Soják) Mosyakin & Shiyan	-	-	-	-	-	-	+	-	-
Spiraea crenata L.	-	+	-	-	-	-		-	-
S. hypericifolia L.	+	-	+	+	-	-	+	+	+
Rubiaceae Juss.									

Galium verticillatum Danthoine ex Lam.	-	-	-	+	-	-	-	-	-
Rutaceae Juss.									
Haplophyllum acutifolium (DC.) G.Don	-	-	-	+	-	-	-	+	+
H. latifolium Kar. & Kir.	-	-	-	-	-	-	-	+	+
* *H. multicaule* Vved.	-	+	-	-	-	-	-	-	-
Thymelaeaceae Juss.									
Diarthron ammodendron (Kar. & Kir.) ined.	+	-	-	-	-	-	-	-	-

Note. Sources of information: 1 – Suvorov, Shilina, 1955; 2 – Rusanov, 1961; Fisyun, 1982; 4 – Khramtsov, 1983; 5 – Baitulin, Sinitsyna, 1991; 6 – Kokoreva, Danilov, 2006; 7 – Kokoreva et al., 2013; 8 – Kudabaeva et al., 2015, including stock materials of the Institute of Botany and Phytointroduction; 9 – Tarnakty, Shilozek, 2023.
* endemic of Kazakhstan; [+] included into the Red Data Book of Kazakhstan (2014).
