# Supplementary figures and images for: Habitats, Plant Diversity, and Molecular Phylogeny of Endemic Relic Species Incarvillea semiretschenskia (Bignoniaceae)
Source: Plants (Basel). 2024 Nov 23;13(23):3299. doi: 10.3390/plants13233299 (PMC11644528; doi:10.3390/plants13233299)

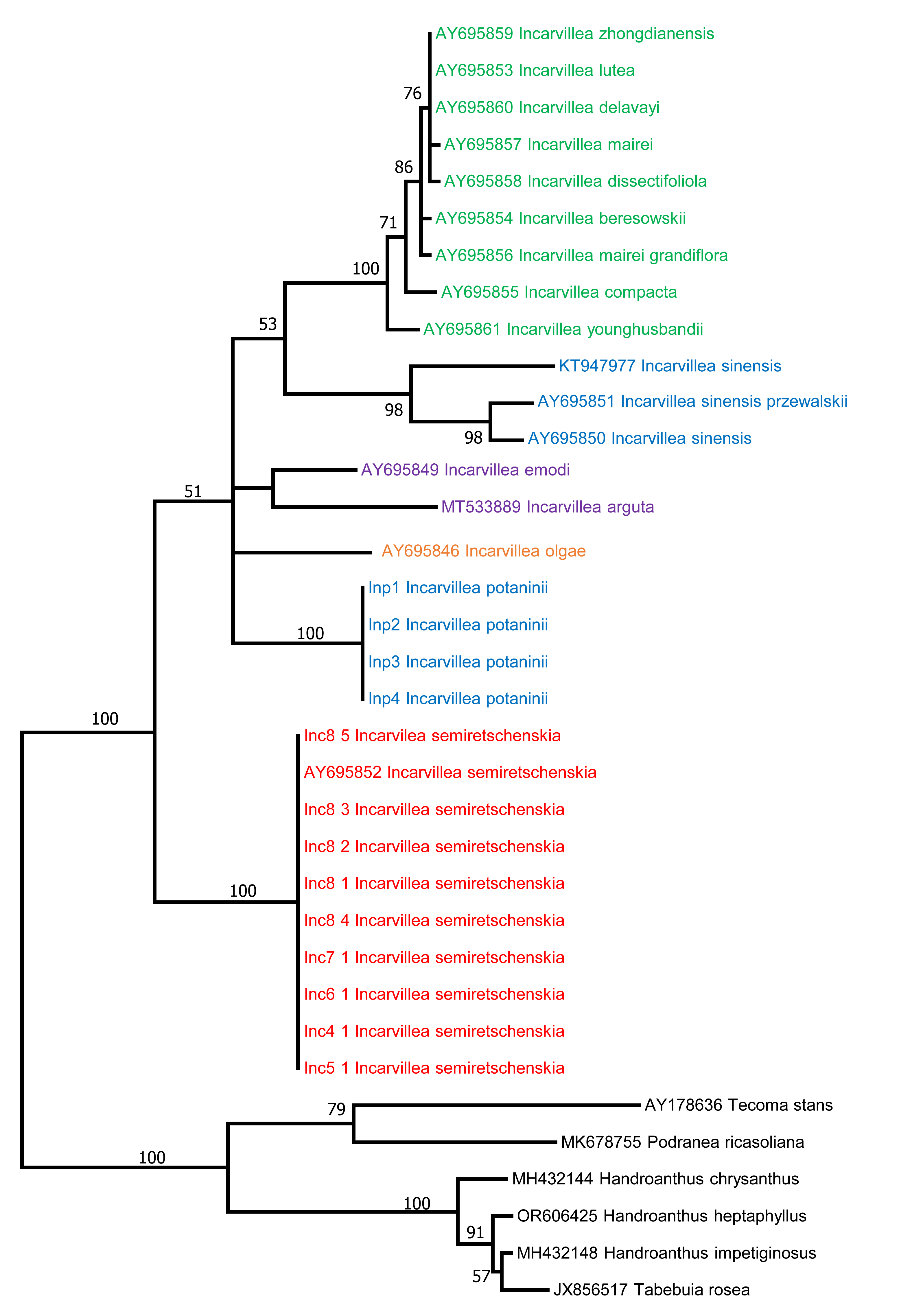

Supplement: Supplementary file 1 [file plants-13-03299-s001.zip › Figure S1 Incarvillea ITS ML tree.jpg]

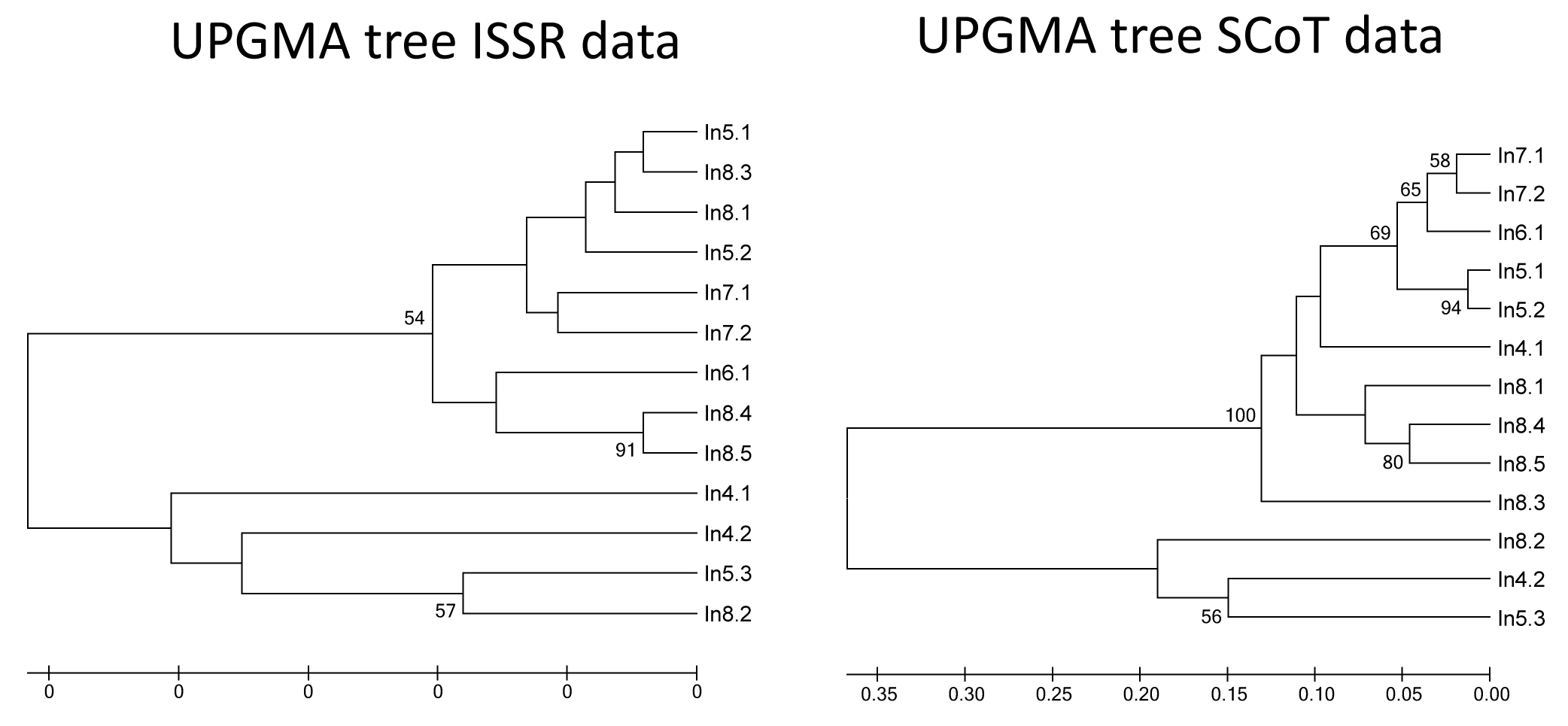

Supplement: Supplementary file 1 [file plants-13-03299-s001.zip › Figure S2. Incarvillea ISSR SCoT UPGMA.jpg]

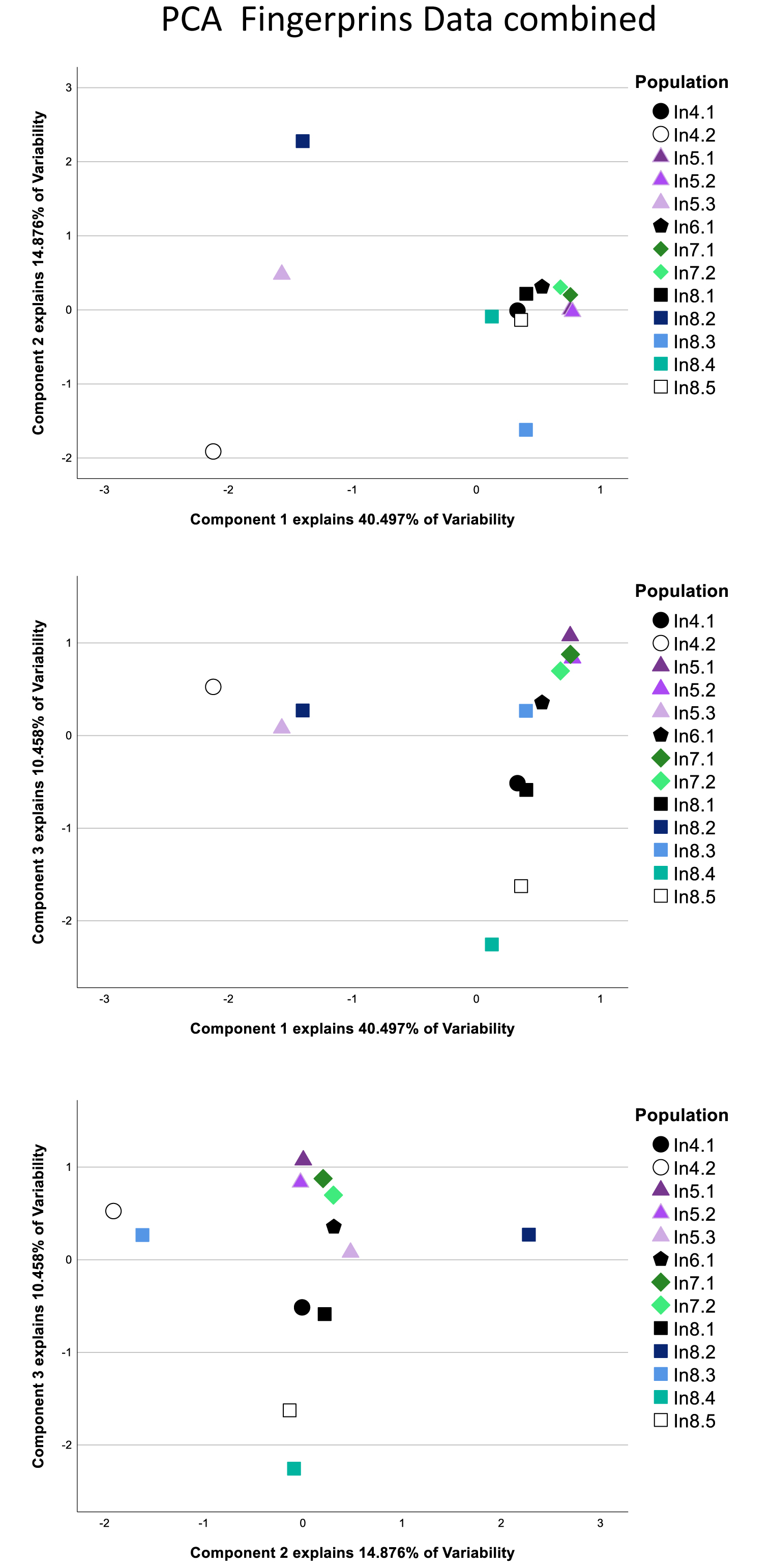

Supplement: Supplementary file 1 [file plants-13-03299-s001.zip › Figure S3. Incarvillea PCA analyse.jpg]
